# Understanding the impact of covariates for trachoma prevalence prediction using geostatistical methods

**DOI:** 10.1186/s44263-025-00161-x

**Published:** 2025-06-01

**Authors:** Misaki Sasanami, Ibrahim Almou, Adam Nouhou Diori, Ana Bakhtiari, Nassirou Beidou, Donal Bisanzio, Sarah Boyd, Clara R. Burgert-Brucker, Abdou Amza, Katherine Gass, Boubacar Kadri, Fikreab Kebede, Michael P. Masika, Nicholas P. Olobio, Fikre Seife, Abdoul Salam Youssoufou Souley, Amsayaw Tefera, Amir B. Kello, Anthony W. Solomon, Emma M. Harding-Esch, Emanuele Giorgi

**Affiliations:** 1 Lancaster Medical School, Lancaster University, Lancaster, UK; 2 Ministère de La Santé, Programme National de Santé Oculaire, Niamey, Niger; 3 Faculty of Health Sciences, Abdou Moumouni University of Niamey, Niamey, Niger; 4 International Trachoma Initiative, Task Force for Global Health, Decatur, GA USA; 5 Ministère de La Santé, National Blindness Prevention Program, Niamey, Niger; 6 RTI International, Washington, DC USA; 7 Clinical Research Department, London, School of Hygiene & Tropical Medicine, London, UK; 8 The Task Force for Global Health, Decatur, GA USA; 9 Ministry of Health, Addis Ababa, Ethiopia; 10 Ministry of Health, Lilongwe, Malawi; 11 Federal Ministry of Health, Abuja, Nigeria; 12 Ophtalmologie de L’Hôpital Amirou Boubacar Diallo de Niamey, Niamey, Niger; 13 World Health Organization Regional Office for Africa, Brazzaville, Congo; 14 Global Neglected Tropical Diseases Programme, World Health Organization, Geneva, Switzerland

**Keywords:** Covariates, Disease mapping, Evaluation unit, Geostatistics, Neglected tropical diseases, Trachoma

## Abstract

**Background:**

Model-based geostatistics (MBG) is increasingly used for estimating the prevalence of neglected tropical diseases, including trachoma, in low- and middle-income countries. We sought to investigate the impact of spatially referenced covariates to improve spatial predictions for trachomatous inflammation—follicular (TF) prevalence generated by MBG. To this end, we assessed the ability of spatial covariates to explain the spatial variation of TF prevalence and to reduce uncertainty in the assessment of TF elimination for pre-defined evaluation units (EUs).

**Methods:**

We used data from Tropical Data-supported population-based trachoma prevalence surveys conducted in EUs in Ethiopia, Malawi, Niger, and Nigeria between 2016 and 2023. We then compared two models: a model that used only age, a variable required for the standardization of prevalence as used in the routine, standard prevalence estimation, and a model that included spatial covariates in addition to age. For each fitted model, we reported estimates of the parameters that quantify the strength of residual spatial correlation and 95% prediction intervals as the measure of uncertainty.

**Results:**

The strength of the association between covariates and TF prevalence varied within and across countries. For some EUs, spatially referenced covariates explained most of the spatial variation and thus allowed us to generate predictive inferences for TF prevalence with a substantially reduced uncertainty, compared with models without the spatial covariates. For example, the prediction interval for TF prevalence in the areas with the lowest TF prevalence in Nigeria narrowed substantially, from a width of 2.9 to 0.7. This reduction occurred as the inclusion of spatial covariates significantly decreased the variance of the spatial Gaussian process in the geostatistical model. In other cases, spatial covariates only led to minor gains, with slightly smaller prediction intervals for the EU-level TF prevalence or even a wider prediction interval.

**Conclusions:**

Although spatially referenced covariates could help reduce prediction uncertainty in some cases, the gain could be very minor, or uncertainty could even increase. When considering the routine, standardized use of MBG methods to support national trachoma programs worldwide, we recommend that spatial covariate use be avoided.

**Supplementary Information:**

The online version contains supplementary material available at 10.1186/s44263-025-00161-x.

## Background

Trachoma is one of twenty-one neglected tropical diseases (NTDs) and is targeted for elimination as a public health problem by 2030 [1, 2]. It is the leading infectious cause of blindness and is known to be a public health problem in 39 countries [3]. The causative agent, *Chlamydia trachomatis* (*Ct*), is transmitted mainly by eye-seeking flies (*Musca sorbens*), direct contact with an infected person, and fomites such as shared towels, bedding, or hard surfaces [4]. Ocular *Ct* infection results in inflammation of the conjunctiva, wherein some individuals can exhibit trachomatous inflammation—follicular (TF), a sign of “active trachoma” [4, 5]. After years of repeated infections, severe inflammation can lead to visible scarring in the upper eyelid conjunctiva. Subsequently, the eyelid can turn inward such that the eyelashes touch the eyeball [4]. This condition is referred to as trachomatous trichiasis (TT) [5], a painful condition that can result in corneal opacity and blindness if not treated.

The focal nature of trachoma has been demonstrated in the clustering of cases at various spatial scales from the household to the district level [4]. This clustering pattern indicates that transmission dynamics and factors associated with trachoma operate at multiple spatial levels, highlighting the importance of avoiding analyses that aggregate data across too large an area. Such aggregation could obscure local variations and lead to over-smoothing of prevalence estimates. By accounting for these localized variations, targeted interventions can be more effectively designed and implemented. A recent systematic review identified that at the community or district level, the main factors associated with trachoma can be categorized as demographic, infrastructural, climatic, and environmental [6]. This review found that factors associated with TF were mean annual precipitation, mean annual temperature, altitude, ruralness, accessibility, access to medical services and schools, and access to water and sanitation. Limited access to water and sanitation could negatively impact the hygiene practices of individuals and increase the presence of *M. sorbens*, which prefers to breed in human feces left exposed to the soil. The mechanism that links climate and environment to *Ct* infections is less clear. Previous studies, which were reviewed in the systematic review, have argued that climate factors, including precipitation and temperature, can affect the life cycle and survival of *M. sorbens*, conjunctival dryness, and irritation due to low humidity, water availability, agricultural productivity, and livelihoods, whereas environmental factors, such as altitude, might be linked to population density, temperature, and socio-economic status in some contexts [7–17]. Regarding other demographic and infrastructural factors, rural areas tend to have worse socioeconomic and sanitation conditions [18] and are remote from services including water and sanitation [4]. Furthermore, lower access to healthcare may also indicate worse socioeconomic development and lower standards of living [19]. Better access to schools might be linked to better health literacy, as parents’ educational attainment can have a protective effect on children’s risk of active trachoma (TF and trachomatous inflammation—intense, TI) [20].

Mapping of disease burden is one of the key components of NTD programs; it guides eradication, elimination, and control efforts [1]. For trachoma, endemic countries have conducted prevalence surveys following the globally standardized survey methods set by the World Health Organization (WHO) and implemented by the Global Trachoma Mapping Project and its successor Tropical Data [21, 22]. Using these data, countries’ elimination status is assessed against the prevalence-based elimination criteria defined by WHO, which are a prevalence of TT unknown to the health system < 0.2% in adults aged ≥ 15 years and a prevalence of TF < 5% in children aged 1–9 years. These criteria are assessed in formerly endemic evaluation units (EUs), which are defined as the administrative units for health care management, typically containing 100,000 to 250,000 persons [23].

One of the endgame challenges for the elimination of NTDs is that it becomes more difficult to precisely predict the disease burden because of the small number of cases relative to survey sample sizes. For NTDs, this is a particular problem because survey data are often spatially sparse due to resource constraints and the inaccessibility of some geographical areas. To address these challenges, model-based geostatistics (MBG) [24] has been increasingly used for mapping NTD risk and for the design of prevalence surveys [25, 26]. One of the key advantages of MBG is that it enables users to borrow information across space, reducing the uncertainty inherent to inferences on disease risk at any desired location within a study area of interest. It has been shown that MBG can produce more precise prevalence estimates than the standard design-based approach for analyzing TT data from trachoma prevalence surveys [27], and its statistical modeling framework applies to a wide range of settings for TF and TT [28].

Although the previous application of geostatistical models to trachoma accounted for individual-level factors associated with trachoma, namely, age in the case of TF and age plus gender for TT [27, 28], the usefulness of the inclusion of spatially referenced associated factors (or covariates) for the prevalence prediction has not been fully investigated. The use of spatial covariates related to environmental and socio-demographic factors for trachoma could enhance the predictive performance of geostatistical models. This is because the use of spatially referenced covariates can help explain the spatial variation in disease prevalence and relies less on residual spatial correlation for carrying out spatial predictions. However, in the context of trachoma elimination, the low prevalences could make the estimation of regression coefficients more difficult to recover from the data and thus limit the usefulness of spatial covariates.

Given that the relationship between covariates and TT is likely to be weaker than for TF because TT prevalence reflects historical rather than current transmission, for this initial analysis, we aimed to examine the impact of spatially referenced covariates on TF prevalence prediction, analyzing trachoma prevalence survey data from Ethiopia, Malawi, Niger, and Nigeria. To this end, we compared models with and without spatially referenced covariates with respect to the following two metrics: the parameter estimates of the geostatistical models, and the predicted TF prevalence and 95% prediction intervals, to summarize the contribution of spatial covariates to model variation in TF prevalence.

## Methods

### Data

#### Trachoma prevalence survey data and selection of evaluation units

We analyzed available data from Tropical Data-supported population-based trachoma prevalence surveys conducted in Ethiopia, Malawi, Niger, and Nigeria. Surveys were implemented based on a two-stage cluster sampling methodology, which is described in detail elsewhere [21, 22, 29, 30]. We used data from the most recent survey for each EU in the country.

To cover different levels of trachoma transmission, we analyzed the EUs that had the lowest, median, and highest observed prevalence of TF as EUs of interest in the country. This selection was made to better understand how the effects of covariates vary under different transmission levels. We then combined the data from contiguous EUs, if available, which were identified as the sampled cluster locations that fell within the most recently available polygon shapefile for those EUs at the time of the analysis (May 2023) [31]. If (1) a geostatistical model was not feasible or (2) the corresponding information on their geographical boundaries was not available in the shapefile, we then instead selected as the EU of interest one that had the closest prevalence level to the one that was originally chosen for the analysis.

The prevalence data used for the analyses in this paper are owned by the governments of the countries in which the original surveys were conducted. Permission to use the data for our analyses was obtained via formal agreements between those governments and the involved universities and organizations; all data were de-identified before use. Researchers are welcome to request access to the same de-identified datasets by contacting Tropical Data at support@tropicaldata.org.

#### Identifying data scenarios suitable for geostatistical modeling

In this study, we defined “a geostatistical model was not feasible” to be when a model could not be fitted, or the 95% confidence intervals of the estimated parameters were not sensible. Examples of the latter case included the intervals that ranged from 0 to infinity or the intervals for the estimated scale of the spatial correlation being so small or large that the lower or upper limit, respectively, were less or more than the minimum or maximum distance between the investigated clusters. Henceforth, we shall refer to each of the datasets identified for each country as “lowest,” “median,” and “highest” TF prevalence EUs, respectively. We excluded data from baseline surveys (i.e., pre-intervention data) and analyzed trachoma impact and/or trachoma surveillance surveys (i.e., post-intervention data).

#### Spatially referenced covariate data

We obtained the data for 18 spatially referenced candidate covariates (Table 1 and Additional file 1: Table S1 for more detailed information), which we considered the most relevant to TF prevalence based on the existing evidence [6] and on consultation with experts in trachoma epidemiology. We then classified these spatial covariates into five categories, each of which was considered to have a primary impact on TF epidemiology. The five categories were environment; accessibility; water, sanitation, and hygiene (WASH); accessibility to and acceptance of health services; and ruralness. For Niger, except for the proportion of the population using insecticide-treated nets, the data on WASH and accessibility to and acceptance of health services were unavailable. The spatial covariates’ values were rescaled to and extracted at the spatial resolution of 5 km^2^ for each cluster location and year (if possible) of the surveys for the EUs of interest. We deemed a 5 km^2^ spatial resolution appropriate for this study, as some modeled covariate data may not accurately represent values at finer spatial scales. Where the data were missing, we interpolated them by taking the mean of the neighborhood of the focal pixel using the R package “terra” [32]. However, for the temperature data, the pixels with missing values were too extensively distributed spatially, so we instead interpolated them using a time series model, which is described in detail in Additional file 2. After the extraction of all spatial covariate values, the continuous variables were standardized such that they had zero mean and one standard deviation. For a categorical variable (e.g., ruralness), we categorized the value into two, representing rural and urban as per a pre-defined classification [33].

**Table 1:** List of spatially referenced candidate covariate

Category	Spatially referenced covariate	Reference
Environment	Precipitation	[34]
	Temperature	[35, 36]
	Enhanced vegetation index (EVI)	[37, 38]
	Aridity index	[39, 40]
	Altitude	[41]
Accessibility	Travel time to cities	[42]
	Travel time to healthcare (motorized and walking only)	[43]
	Distance to OpenStreetMap (OSM) major roads	[41, 44]
	Distance to OSM major waterways	[41, 44]
Water, sanitation and hygiene (WASH)	Percentage of population using an improved water source	[45, 46]
	Percentage of the population using open defecation	[45, 46]
Accessibility to and acceptance of health services	Percentage of children receiving at least one dose of diphtheria, tetanus toxoid, and pertussis (DPT) vaccine	[45, 46]
	Percentage of children receiving measles vaccination	[45, 46]
	Percentage of live births delivered at a health facility	[45, 46]
	Proportion of population using insecticide-treated nets	[47, 48]
Ruralness	Nighttime lights	[49–51]
	Population density	[41, 52]
	Ruralness	[33]

#### Census data

We used population census data from each country to produce the age-standardized TF prevalence. The proportion of each 1-year age band among children aged 1–9 years was calculated based on the 2007 Population and Housing Census in Ethiopia [53], the 2018 Population and Housing Census in Malawi [54], the General Census of Population and Housing 2012 in Niger [55], and the 2006 Population and Housing Census in Nigeria [56].

#### Population density data

To weight the predicted TF prevalence by the population density for each prediction location in Malawi, we collected the data on the population density of 1–9-year-olds for the year 2020 obtained from WorldPop [57]. For Ethiopia, Niger, and Nigeria, the general population was used, because the data relating to 1–9-year-olds had many missing values. As the datasets for the general population were available for the years from 2000 to 2020, we used the data for the year during which the trachoma prevalence surveys were conducted in each EU of interest, or for the year 2020, if the survey was carried out after 2020. The use of the population density data is required to weigh pixels at the prediction stage of the analysis, which is not otherwise possible with the census data as these are not available at a fine spatial resolution. This aspect will be further explained in the “Prediction” section of the “Methods” section.

### Statistical modeling

#### Accounting for age effects and spatial covariates selection

To build a geostatistical model to predict the TF prevalence using each dataset for the lowest, median, and highest TF prevalence EUs identified in the previous section, we followed the general guidance for geostatistical modeling with spatially referenced covariates which was outlined by Giorgi et al. [58] and aimed to maximize the predictive power, whilst maintaining reasonable explanatory power by selecting and categorizing the candidate spatial covariates based on existing scientific knowledge as described above [58, 59].

Table 2 provides a list of all the statistical models considered in the study, for which we now provide further details. We first developed a model including age and retained it regardless of its statistical significance, following the modeling framework shown in the previous study [28]. Hence, we used a linear spline with a knot between ages 2 and 4 years to model the effects of age on the logit TF prevalence if the observed prevalence showed an increase until around age 2–4 years and a decrease afterwards (model 1). The peak age, where the knot was placed, was informed by eye, through inspection of plotted data. If the empirical logit prevalence instead linearly increased with increasing age, we modeled the age effects as logit linear accordingly. Formally,

1 $$log\left\{\frac{{p}_{j}\left({x}_{i}\right)}{1-{p}_{j}\left({x}_{i}\right)}\right\}= \alpha +\mathcal{f}\left({d}_{ij}\right).$$

2 $$f\,\left(d_{ij}\right)=\left\{\begin{array}{l}\beta_1d_{ij}+\beta_2max\left(d_{ij}-m,0\right)\;\text{if }2\leq m\leq4\\ \quad\quad\ \ \beta_1d_{ij}\qquad\qquad\qquad \, \text{otherwise}\end{array}.\right.$$

where ${p}_{j}\left({x}_{i}\right)$ is the probability that a child $j$ living in the cluster ${x}_{i}$ has TF. $\alpha$ is an intercept. $d$ represents age, and $\beta$ is a regression coefficient. $m$ represents the age at which the observed prevalence is the highest.

**Table 2:** List of the models considered in this study

Model	Type of model	Covariates
Model 1	GLM	Age only
Model 2	GLM	Spatially referenced covariates and age
Model 3	GLMM	Spatially referenced covariates and age
Model 4	Geostatistical model	Spatially referenced covariates and age

We then included spatially referenced covariates as follows:

3 $$log\left\{\frac{{p}_{j}\left({x}_{i}\right)}{1-{p}_{j}\left({x}_{i}\right)}\right\}= \alpha +\mathcal{f}\left({d}_{ij}\right)+e\left({x}_{i}\right){\prime}\gamma .$$

where $e\left({x}_{i}\right)$ is the spatially referenced covariates and $\gamma$ is a regression coefficient.

**Fig. 1:**
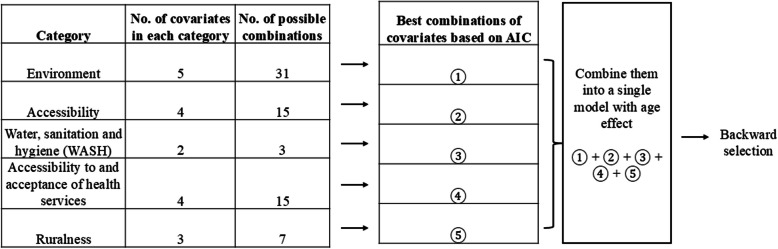
Selection process of spatially referenced covariates. Within each spatial covariate category, all the possible combinations of variables were examined using a generalised linear model (GLM) [60, 61] if the variables were entered non-linearly), and the ones that produced the lowest Akaike Information Criterion (AIC) [62] were selected. These covariates were then combined into a single model, followed by a backward selection process [63, 64]

To build model 2, for each dataset identified as lowest, median, and highest TF prevalence EUs, we first visually assessed the linear and nonlinear association between the empirical logit prevalence of TF at cluster level among children aged 1–9 years and each of the spatial covariates by fitting univariate models. Based on the visual assessment, for each dataset, we determined whether the spatial covariates needed to be log-transformed to render the association with the logit prevalence more linear, and whether, after taking the log, the resulting relationship could be assumed linear or required additional adjustments due to nonlinear effects. We then examined all possible combinations of spatial covariates within each of the five categories using a generalized linear model (GLM) [60, 61]. Among these combinations, we selected those that produced the lowest Akaike information criterion (AIC) [62]. The five groups of variables were then merged into a single model including the age effect, and we took a backward elimination process until the removal of the remaining variables no longer reduced AIC [63, 64]. The process of spatial covariate selection is illustrated in Fig. 1.

To account for extra binomial variation due to within-cluster correlation, we expanded Model 2 into a generalized linear mixed model (GLMM), where we introduced independent and identically distributed Gaussian random noise (Model 3). In this study, we fixed the effects of the selected covariates as an offset as follows:

4 $$log\left\{\frac{{p}_{j}\left({x}_{i}\right)}{1-{p}_{j}\left({x}_{i}\right)}\right\}=\widehat{\alpha }+\widehat{\mathcal{f}}\left({d}_{ij}\right)+e\left({x}_{i}\right){\prime}\widehat{\gamma }+{Z}_{i}.$$

where $\widehat{\alpha }$, $\widehat{\mathcal{f}}$, and $\widehat{\gamma }$ are the offset for which we used the parameter estimates in model 2. ${Z}_{i}$ is independent and identically distributed Gaussian noise with mean zero and variance ${\tau }^{2}$, representing the residual variation in prevalence ${p}_{j}\left({x}_{i}\right)$ that was not attributable to the covariates.

Since the random effects in model 3 should be independent, if they are spatially correlated, that would invalidate the use of a GLMM. In such cases, we used a geostatistical model (model 4) which we defined as follows:

5 $$log\left\{\frac{{p}_{j}\left({x}_{i}\right)}{1-{p}_{j}\left({x}_{i}\right)}\right\}=\widehat{\alpha }+\widehat{\mathcal{f}}\left({d}_{ij}\right)+e\left({x}_{i}\right){\prime}\widehat{\gamma }+S\left({x}_{i}\right)+{Z}_{i}.$$

where $S\left(x\right)$ is a spatial Gaussian process that has mean zero, variance ${\sigma }^{2}$, and correlation function $\rho \left(u\right)=Corr\left(S\left(x\right),S\left({x}{\prime}\right)\right)=exp\left\{-\left|x - {x}{\prime}\right|/\phi \right\}$, where $\phi$ is the decay rate of spatial correlation as the distance increases between $x$ and ${x}{\prime}$. Note that the model fitting process allowed for the data to determine the scale of the spatial correlation rather than us imposing any presumption on it. The parameters of the model were estimated by Monte Carlo maximum likelihood (MCML) based on the R package “PrevMap” [65].

The type of model is either a generalized linear model (GLM), generalized linear mixed model (GLMM), or geostatistical model.

#### Prediction

Using the geostatistical model (model 4), we predicted the TF prevalence as follows. We first predicted the age-specific prevalence, ${P}_{a}$ at locations ${x}^{*}$ within the EU of interest at 5 km^2^ spatial resolution. We then computed the age-standardized prevalence, $P$, using the population census data, i.e.,

6 $${P}_{a}=\left\{{P}_{a}\left( {x}^{*}\right): {x}^{*}\in \text{EU}\right\}$$

7 $$P\left({x}^{*}\right)=\sum\limits_{a=1}^{9}{\mathcal{W}}_{a}\left({x}^{*}\right){P}_{a}\left({x}^{*}\right)$$

where $a$ represents the 1-year age band of children aged 1–9 years and ${\mathcal{W}}_{a}\left( {x}^{*}\right)$ is the 1 proportion of the population in each age group $a$ at the location ${x}^{*}$.

To compute the EU-wide prevalence, we gave the prevalence weights of the population density, $Pop$ i.e.,

8 $$P\left(EU\right)=\frac{\sum_{\mathcal{x}\mathcal{*}\in EU}P\left(\mathcal{x}\mathcal{*}\right)Pop\left({x}^{*}\right)}{\sum_{\mathcal{x}\mathcal{*}\in EU}Pop\left({x}^{*}\right)}$$

We obtained 10,000 predictive samples of $S\left({x}^{*}\right)$ by MCML. Using these samples, we computed the following two quantities: the mean as the point prediction of the EU-wide standardized prevalence; and the 2.5 th and 97.5 th range as the 95% prediction interval.

When the GLMM (model 3) was used as a final model, we followed the same approach for age standardization and weighting by population density. We obtained 10,000 predictive samples from $Z\left({x}^{*}\right)$ by MCML and then computed the abovementioned two quantities.

#### Spatially referenced covariates’ impact assessment by model comparison

To assess the impact of spatially referenced covariates, we compared the following two metrics. First, we compared the estimates of the covariance parameters of the geostatistical model with and without the spatial covariates, with the aim of understanding whether the spatial covariates explained the spatial correlation in the TF prevalence, and if so at what spatial scale. Second, we compared the predicted prevalence and the 95% prediction intervals for each EU of interest to investigate whether the inclusion of spatial covariates reduced the uncertainty in the prevalence prediction.

## Results

We analyzed data from a total of 12 EUs. Table 3 shows the crude (unadjusted) TF prevalence among children aged 1–9 years for the lowest, median, and highest TF prevalence EUs in Ethiopia, Malawi, Niger, and Nigeria. The maps of analyzed EUs are presented in Additional file 1: Figure S1–4. Overall, Ethiopia had higher prevalences than the other countries. In all countries, most of the EUs of interest were surrounded by areas that had noticeably different prevalence.

**Table 3:** Crude (unadjusted) prevalence of trachomatous inflammation—follicular (TF) among children aged 1–9 years. The EUs of interest in Ethiopia, Malawi, Niger, and Nigeria were those that had the lowest, median, and highest TF prevalence in the most recent available dataset, and the contiguous areas represent the adjacent areas to each EU of interest

TF prevalence level	Area type	Ethiopia			Malawi			Niger			Nigeria		
		Crude prevalence (%)	Year	Type	Crude prevalence (%)	Year	Type	Crude prevalence (%)	Year	Type	Crude prevalence (%)	Year	Type
Lowest EUs	EU of interest	0.17 (2/1,150)	2021	TIS	0.76 (6/794)	2019	TSS	0.19 (3/1,572)	2022	TSS	0.05 (1/1,824)	2022	TSS
	Contiguous areas	2.63 (144/5,474)	2021–22	TIS	0.01 (61/4,469)	2018, 2019	TSS	2.53 (330/13,051)	2018–19, 2021–22	TIS, TSS	2.64 (263/9,951)	2019, 2021–22	TIS, TSS
Median EUs	EU of interest	6.47 (64/989)	2022	TSS	1.54 (14/912)	2019	TSS	1.98 (22/1,113)	2018	TSS	1.01 (17/1,689)	2020	TSS
	Contiguous areas	6.46 (428/6,622)	2018–22	TIS, TSS	1.29 (73/5,646)	2018, 2019	TSS	2.12 (86/4,049)	2018	TSS	3.24 (248/7,644)	2019–20, 2022	TIS, TSS
Highest EUs	EU of interest	55.31 (401/725)	2022	TIS	5.00 (55/1,099)	2017	TSS	5.95 (130/2,184)	2022	TSS	12.71 (192/1,511)	2018	TIS
	Contiguous areas	14.55 (596/4,095)	2019, 2021–22	TIS, TSS	1.59 (32/2,014)	2017, 2019	TSS	3.17 (578/18,214)	2019, 2021–22	TIS, TSS	1.59 (84/5,274)	2018, 2021	TIS, TSS

Year and type indicate the year and the type of the survey, where TIS and TSS, respectively, represent the trachoma impact survey and trachoma surveillance survey.

The parameter estimates for the covariates, and the transformations, if any, that were applied are shown in Additional file 1: Table S2–13. We found that the selected covariates differ across and even within the countries. For example, in Malawi, five spatial covariates were selected from the categories of accessibility, WASH, and ruralness in the median TF prevalence EUs, whereas for the highest TF prevalence EUs, seven covariates were selected from the categories of environment and accessibility. The effects of covariates on trachoma prevalence varied across countries and EUs, reflecting differences in environmental conditions, accessibility, and socio-demographic factors. For example, in Ethiopia, a higher aridity index was associated with increased odds of trachoma in lowest-prevalence areas (OR 4.17, 95% CI 3.10–5.63) but showed a protective effect in highest-prevalence areas (OR 0.45, 95% CI 0.33–0.61). Similarly, in Niger, precipitation had a strong negative association with trachoma in median-prevalence areas (OR 0.08, 95% CI 0.03–0.24) but was positively associated with prevalence in high-burden areas (OR 1.38, 95% CI 1.15–1.66). Accessibility also showed contrasting effects; in Malawi, increased travel time to cities was associated with higher odds of trachoma in highest-prevalence areas (OR 2.20, 95% CI 1.59–3.04), whereas in Nigeria, longer travel time to cities was linked to lower odds in lowest-prevalence areas (OR 0.49, 95% CI 0.36–0.67). These findings highlight the importance of accounting for local variations in factors associated with TF when modeling TF prevalence.

Table 4 shows the estimated covariance parameters in the geostatistical models with and without spatially referenced covariates. In some cases, the variance of the Gaussian noise, ${\tau }^{2}$, was not able to be estimated and was therefore removed from the model. Where the results are not presented, the GLMM was used as the final model because there was no residual spatial correlation. We see that, in eight out of 12 cases, the inclusion of the spatial covariates simplified the model from a geostatistical model to a GLMM. Where geostatistical models were still used after accounting for the covariates, the estimated values for the variance ${\sigma }^{2}$ and the spatial scale parameter $\phi$ of the spatial Gaussian process became smaller. There was a substantial reduction in the variance in the lowest TF prevalence EUs in Nigeria by about 7, and in the lowest TF prevalence EUs in Niger, the spatial scale parameter fell by approximately 80, although the confidence intervals between the models overlapped.

**Table 4:** The parameter estimates and 95% confidence intervals (CI) of the geostatistical model

Country	Parameter	OR estimates (95% CI)					
		Lowest TF prevalence EUs		Median TF prevalence EUs		Highest TF prevalence EUs	
		With covariates	Without covariates	With covariates	Without covariates	With covariates	Without covariates
Ethiopia	${\sigma }^{2}$	1.11 (0.64, 1.99)	3.84 (1.87, 7.88)	-	1.31 (0.66, 2.56)	-	2.33 (1.03, 5.24)
	$\phi$	1.54 (0.71, 3.33)	18.09 (8.63, 38.17)	-	18.29 (7.12, 45.53)	-	18.65 (5.37, 64.02)
	${\tau }^{2}$	-	-	-	0.48 (0.21, 1.08)	-	0.44 (0.18, 1.06)
Malawi	${\sigma }^{2}$	-	0.01 (0.01, 0.021)	-	0.11 (0.06, 0.18)	-	0.63 (0.19, 2.09)
	$\phi$	-	7.02 (3.71, 13.39)	-	5.79 (2.65, 12.35)	-	18.93 (3.86, 93.19)
	${\tau }^{2}$	-	-	-	-	-	-
Niger	${\sigma }^{2}$	2.02 (0.99, 4.13)	7.06 (2.77, 18.24)	-	3.63 (1.52, 8.66)	-	0.76 (0.37, 1.56)
	$\phi$	26.47 (12.63, 56.20)	106.43 (47.19, 246.12)	-	23.70 (10.05, 57.49)	-	20.81 (6.20, 66.24)
	${\tau }^{2}$	0.11 (0.03, 0.49)	0.18 (0.06, 0.48)	-	-	-	0.43 (0.21, 0.87)
Nigeria	${\sigma }^{2}$	1.44 (1.12, 1.86)	8.82 (4.43, 17.24)	-	3.38 (1.89, 6.18)	1.15 (0.27, 4.85)	4.69 (1.87, 12.26)
	$\phi$	0.90 (0.42, 1.99)	15.22 (7.81, 29.47)	-	6.96 (3.75, 13.07)	6.88 (1.60, 29.99)	15.99 (5.41, 47.53)
	${\tau }^{2}$	-	-	-	-	0.26 (0.01, 5.80)	-

The models with and without spatially referenced covariates are compared for the lowest, median, and highest trachomatous inflammation—follicular (TF) prevalence evaluation units (EUs) in Ethiopia, Malawi, Niger, and Nigeria. The parameter estimates and CIs are shown on the odds ratio (OR) scale. ${\sigma }^{2}$ and $\phi$, respectively, correspond to the variance and the scale of the exponential spatial correlation of the Gaussian process $S\left({x}_{i}\right)$. ${\tau }^{2}$ is the variance of a non-spatially structured Gaussian random variable (i.e., nugget effect). The results are not shown when the geostatistical model was not able to be fitted.

Table 5 shows the predicted TF prevalence and the 95% prediction interval for the EUs of interest. In all EUs, the predicted prevalences were consistent between the model with and without the spatially referenced covariates, as the 95% prediction interval overlapped between the models. In most EUs, the precision of the prediction improved by accounting for the spatial covariates, narrowing the 95% prediction intervals. However, in the lowest TF prevalence EU in Malawi, uncertainty in the prevalence prediction was increased.

**Table 5:** Predicted trachomatous inflammation—follicular (TF) prevalence (%) in 1–9-year-olds

Country	Predicted TF prevalence (95% prediction interval)					
	Lowest TF prevalence EU of interest		Median TF prevalence EU of interest		Highest TF prevalence EU of interest	
	With covariates	Without covariates	With covariates	Without covariates	With covariates	Without covariates
Ethiopia	0.55 (0.42, 0.72)	1.95 (0.65, 4.48)	14.61 (11.15, 18.50)	8.68 (5.23, 13.13)	42.91 (37.19, 48.59)	49.70 (41.61, 57.60)
Malawi	1.29 (0.95, 1.84)	1.27 (1.15, 1.42)	1.20 (0.96, 1.50)	1.30 (1.04, 1.61)	3.95 (3.36, 4.65)	4.30 (3.18, 5.64)
Niger	1.25 (0.77, 1.92)	1.10 (0.27, 3.23)	1.64 (1.38, 1.95)	1.86 (1.03, 3.17)	6.18 (4.97, 7.53)	6.02 (4.33, 8.20)
Nigeria	1.67 (1.35, 2.05)	1.30 (0.33, 3.20)	0.96 (0.58, 1.57)	1.30 (0.50, 3.44)	9.82 (6.61, 13.81)	9.45 (5.97, 14.22)

The TF prevalence and 95% prediction interval were compared between models with and without spatially referenced covariates for the evaluation units (EUs) of interest in Ethiopia, Malawi, Niger, and Nigeria. The point prevalence and prediction intervals, respectively, were computed as the mean and 2.5 th and 97.5 th range of 10,000 predictive samples based on Monte Carlo maximum likelihood.

## Discussion

In this study, we quantified the inferential benefits that are accrued using spatially referenced covariates in a geostatistical model for trachoma prevalence surveys. Using TF data from Ethiopia, Malawi, Niger, and Nigeria, we have illustrated the geostatistical modeling framework that allowed us to incorporate spatial covariates to predict the TF prevalence of a specific EU and compared those with models that only used the individual-level covariate of age. We found that the spatially referenced covariates helped reduce prediction uncertainty; however, in some cases, the reduction was very small; in a case of very low TF prevalence, the uncertainty in prevalence prediction increased after the inclusion of spatial covariates.

One of the primary challenges encountered in fitting complex models, such as geostatistical models with spatial random effects, was the very low prevalence of TF in several study areas. While our sample sizes were relatively large (as shown in Table 3), the low number of positive cases, in some instances, created difficulties in estimating model parameters with sufficient precision. This issue is not unique to geostatistics; it is a challenge that applies to most complex models when modeling rare events [66–68]. This is because low prevalence leads to sparse signal-to-noise ratios, increasing uncertainty in parameter estimates and limiting the ability to fit highly parameterized models, including geostatistical models. As a result, simpler models may often be more stable and reliable in such settings, as also shown in this study.

We examined the extensive list of factors potentially associated with TF (Table 1 and Additional file 1: Table S1 for more detailed information). In the selection process of spatially referenced covariates for our models, we found that the association between covariates and TF prevalence was substantially different depending on the analyzed EUs, as indicated by the final set of selected covariates and their estimated regression coefficients (Additional file 1: Table S2–13). This suggests that trachoma epidemiology is highly specific to each area, consistent with evidence showing that trachoma is a very focal disease that tends to cluster not only at the household or community level but also at the district level [6, 69]. Therefore, when analyzing data across multiple EUs, careful consideration must be given to potential variations in covariate effects between EUs. As a result, the decision to combine data from multiple contiguous EUs should be made on a case-by-case basis, and one should avoid merging samples from vastly different populations.

We compared the parameters of geostatistical models with and without spatially referenced covariates to assess their impact on modeling spatial variation. In some cases, the inclusion of spatial covariates allowed for the use of simpler GLMMs, indicating that most of the spatial correlation in prevalence was explained by the spatially referenced covariates, leaving no residual spatial correlation in the model. When geostatistical models included spatial covariates, the estimated parameters for the scale of spatial correlation decreased, as the spatial covariates accounted for the large-scale spatial patterns in the data. Additionally, the variance of the spatial Gaussian process decreased, reducing uncertainty in the estimated geostatistical models. Given these findings, in general, the use of GLMMs with spatially referenced covariates should be preferred over geostatistical models without spatial covariates, as these simpler models can provide more precise predictive inferences on disease prevalence, provided that the regression relationships are valid across all locations.

Comparison of the prediction intervals for the estimated TF prevalences based on models with and without spatially referenced covariates showed that in all scenarios, the 95% prediction intervals overlapped between the two models, indicating consistent results. While the inclusion of spatial covariates generally reduced prediction uncertainty, the reduction was minimal in some cases, and uncertainty in fact increased in the lowest TF prevalence EU in Malawi. This increase may be attributed to the low prevalence in the dataset, which includes data from the EU of interest and its surrounding areas, making it more challenging to establish a clear relationship with covariates. Furthermore, unlike other NTDs, the role and impact of the environment in the transmission mechanisms of trachoma are less clear. For example, spatial covariates have been shown to enhance the accuracy and precision of prevalence predictions using geostatistical models for soil-transmitted helminths (STH) [70]. For their transmission, climate with adequate moisture and warm temperature is known to be an important associated factor, as well as inadequate access to water and sanitation, because the parasite eggs or larvae thrive in the warm and moist soil where they infect humans through direct contact or ingestion [71]. In contrast, the transmission of trachoma encompasses direct person-to-person contact and indirect transmission via flies, fomites, or hard surfaces [4, 72–74] and is less well understood in relation to environmental or sociodemographic factors [7–17]. As a result, the spatial distribution of trachoma may not be as effectively explained by the spatial covariates included in the analysis.

When using spatially referenced covariates for prevalence prediction, several considerations are necessary. First, the selection of spatial covariates in the final model may vary depending on how the relationship between covariates and TF prevalence is defined. For example, in this study, we examined the association between annual mean temperature and TF prevalence. However, this relationship may differ if we consider mean temperature on a monthly basis, which could better capture the immediate impact of temperature on the life cycle and density of *M. sorbens*, a key mechanical vector in trachoma transmission [7–17]. It would be, however, very challenging, if not impossible, to discern such effects when dealing with the temporally sparse trachoma prevalence survey data. Similarly, TF prevalence might be driven by past exposures to the associated factors. For instance, one might find a stronger association between the TF prevalence and the precipitation months before the survey. However, to the best of our knowledge, there is no clear evidence to guide the selection of the most appropriate lagged covariate values for investigation. Second, it is important to recognize that covariate data may not perfectly represent the variables of interest, and some of these data are generated from models that are inherently subject to assumptions, biases, and errors. For example, data on healthcare accessibility used in this study were primarily derived from published information on public hospitals and clinics [43]. This could introduce significant bias in areas where private facilities contribute greater proportions of total healthcare delivery. Additionally, the dataset lacks temporal dynamics; as of now, it is only available for the year 2019 and does not account for seasonal or permanent closures of healthcare facilities. Whilst acknowledging these considerations, it is also crucial to emphasize that no causal inferences should be drawn from the covariates used in this study, as our models were designed for prediction and not for the unbiased estimation of regression coefficients.

There are several challenges in applying geostatistical models to trachoma prevalence survey data. Firstly, in some cases, the geographical definitions of EUs on the ground do not correspond with the boundaries represented by the shapefiles. When the geography of the area of interest is not well defined, accurate EU-wide prevalence estimates cannot be produced. Furthermore, in some countries, EU boundaries shift over time due to administrative or political changes, and no official source is available that tracks all changes. As a result, the geographical boundary data for EUs subject to such changes might not be available at the time of analysis. Another challenge is that trachoma surveys are conducted in each EU at different times, based on previous prevalence levels [22] and other programmatic considerations. This means that when data from multiple EUs are combined and analyzed together, as in this study, it is assumed that prevalence levels remain constant throughout the period during which the aggregated data were derived. This assumption overlooks potential temporal changes in prevalence. However, in our study, this was considered reasonable as we excluded baseline survey (i.e., pre-treatment) data from the analysis.

## Conclusions

The association between the spatially referenced covariates and TF prevalence varies both across and within countries. This underscores the highly heterogeneous dynamics of ocular *Ct* infection transmission, and statistical models should be tailored to the contextual setting. Deciding whether or not the spatial covariates should be used would thus have to rely on contextual knowledge and assessment of the scientific validity of the estimated regression relationships with the covariates.

## Supplementary Information

Additional file 1: Provides maps of the evaluation units considered in the analysis,Additional file 1: Provides maps of the evaluation units considered in the analysis, summaries on the clustering of locations across data-sets and tables of the point and interval estimates of the parameters of the geostatistical models. Figure S1. Analysed evaluation units (EUs) in Ethiopia. Figure S2. Analysed evaluation units (EUs) in Malawi. Figure S3. Analysed evaluation units (EUs) in Niger. Figure S4. Analysed evaluation units (EUs) in Nigeria. Figure S5. Average number of observations within a distance radius. Table S1. Data sources of the spatially referenced covariates. Table S2. Parameter estimates and 95% confidence intervals (CI) of the generalised linear model (defined as equation (1) in the manuscript) on the odds ratio (OR) scale for the lowest trachomatous inflammation—follicular (TF) prevalence evaluation units (EUs) in Ethiopia. Table S3. Parameter estimates and 95% confidence intervals (CI) of the generalised linear model (defined as equation (1) in the manuscript) on the odds ratio (OR) scale for the median trachomatous inflammation—follicular (TF) prevalence evaluation units (EUs) in Ethiopia. Table S4. Parameter estimates and 95% confidence intervals (CI) of the generalised linear model (defined as equation (1) in the manuscript) on the odds ratio (OR) scale for the highest trachomatous inflammation—follicular (TF) prevalence evaluation units (EUs) in Ethiopia. Table S5. Parameter estimates and 95% confidence intervals (CI) of the generalised linear model (defined as equation (1) in the manuscript) on the odds ratio (OR) scale for the lowest trachomatous inflammation—follicular (TF) prevalence evaluation units (EUs) in Malawi. Table S6. Parameter estimates and 95% confidence intervals (CI) of the generalised linear model (defined as equation (1) in the manuscript) on the odds ratio (OR) scale for the median trachomatous inflammation—follicular (TF) prevalence evaluation units (EUs) in Malawi. Table S7. Parameter estimates and 95% confidence intervals (CI) of the generalised linear model (defined as equation (1) in the manuscript) on the odds ratio (OR) scale for the highest trachomatous inflammation—follicular (TF) prevalence evaluation units (EUs) in Malawi. Table S8. Parameter estimates and 95% confidence intervals (CI) of the generalised linear model (defined as equation (1) in the manuscript) on the odds ratio (OR) scale for the lowest trachomatous inflammation—follicular (TF) prevalence evaluation units (EUs) in Niger. Table S9. Parameter estimates and 95% confidence intervals (CI) of the generalised linear model (defined as equation (1) in the manuscript) on the odds ratio (OR) scale for the median trachomatous inflammation—follicular (TF) prevalence evaluation units (EUs) in Niger. Table S10. Parameter estimates and 95% confidence intervals (CI) of the generalised linear model (defined as equation (1) in the manuscript) on the odds ratio (OR) scale for the highest trachomatous inflammation—follicular (TF) prevalence evaluation units (EUs) in Niger. Table S11. Parameter estimates and 95% confidence intervals (CI) of the generalised linear model (defined as equation (1) in the manuscript) on the odds ratio (OR) scale for the lowest trachomatous inflammation—follicular (TF) prevalence evaluation units (EUs) in Nigeria. Table S12. Parameter estimates and 95% confidence intervals (CI) of the generalised linear model (defined as equation (1) in the manuscript) on the odds ratio (OR) scale for the median trachomatous inflammation—follicular (TF) prevalence evaluation units (EUs) in Nigeria. Table S13. Parameter estimates and 95% confidence intervals (CI) of the generalised linear model (defined as equation (1) in the manuscript) on the odds ratio (OR) scale for the highest trachomatous inflammation—follicular (TF) prevalence evaluation units (EUs) in Nigeria.

Additional file 2: Provides information on the handling of spatially referenced covariates used in the geostatistical models. Figure S6. Annual average land surface temperature (LST) for the year 2016–22 in Ethiopia. Figure S7. Annual average land surface temperature (LST) for the year 2016–22 in Malawi. Figure S8. Annual average land surface temperature (LST) for the year 2016–22 in Niger. Figure S9. Annual average land surface temperature (LST) for the year 2016–22 in Nigeria. Figure S10. Observed temperature (in Kelvin) and fitted values at randomly selected pixels that contained missing values in Ethiopia . Figure S11. Observed temperature (in Kelvin) and fitted values at randomly selected pixels that contained missing values in Malawi . Figure S12. Observed temperature (in Kelvin) and fitted values at randomly selected pixels that contained missing values in Niger. Figure S13. Observed temperature (in Kelvin) and fitted values at randomly selected pixels that contained missing values in Nigeria.

## Data Availability

The trachoma prevalence survey data from Ethiopia, Malawi, Niger, and Nigeria used in this study are owned by the respective national governments. Access to these data was granted through formal agreements between those governments and the participating universities and organisations. All data were de-identified prior to analysis. Researchers interested in accessing the same de-identified datasets can submit a request to Tropical Data via email at support@tropicaldata.org.
