# Supplementary material for: Understanding the impact of covariates for trachoma prevalence prediction using geostatistical methods
Source: BMC Glob Public Health. 2025 Jun 1;3:48. doi: 10.1186/s44263-025-00161-x (PMC12126867; doi:10.1186/s44263-025-00161-x)
Supplement: Supplementary file 1 — Additional file 1: Provides maps of the evaluation units considered in the analysis,Additional file 1: Provides maps of the evaluation units considered in the analysis, summaries on the clustering of locations across data-sets and tables of the point and interval estimates of the parameters of the geostatistical models. Figure S1. Analysed evaluation units (EUs) in Ethiopia. Figure S2. Analysed evaluation units (EUs) in Malawi. Figure S3. Analysed evaluation units (EUs) in Niger. Figure S4. Analysed evaluation units (EUs) in Nigeria. Figure S5. Average number of observations within a distance radius. Table S1. Data sources of the spatially referenced covariates. Table S2. Parameter estimates and 95% confidence intervals (CI) of the generalised linear model (defined as equation (1) in the manuscript) on the odds ratio (OR) scale for the lowest trachomatous inflammation—follicular (TF) prevalence evaluation units (EUs) in Ethiopia. Table S3. Parameter estimates and 95% confidence intervals (CI) of the generalised linear model (defined as equation (1) in the manuscript) on the odds ratio (OR) scale for the median trachomatous inflammation—follicular (TF) prevalence evaluation units (EUs) in Ethiopia. Table S4. Parameter estimates and 95% confidence intervals (CI) of the generalised linear model (defined as equation (1) in the manuscript) on the odds ratio (OR) scale for the highest trachomatous inflammation—follicular (TF) prevalence evaluation units (EUs) in Ethiopia. Table S5. Parameter estimates and 95% confidence intervals (CI) of the generalised linear model (defined as equation (1) in the manuscript) on the odds ratio (OR) scale for the lowest trachomatous inflammation—follicular (TF) prevalence evaluation units (EUs) in Malawi. Table S6. Parameter estimates and 95% confidence intervals (CI) of the generalised linear model (defined as equation (1) in the manuscript) on the odds ratio (OR) scale for the median trachomatous inflammation—follicular (TF) prevalence e [file 44263_2025_161_MOESM1_ESM.docx]

**Understanding the impact of covariates for trachoma prevalence prediction using geostatistical methods**

Additional file 1

Figure S1. Analysed evaluation units (EUs) in Ethiopia

Figure S2. Analysed evaluation units (EUs) in Malawi

Figure S3. Analysed evaluation units (EUs) in Niger

Figure S4. Analysed evaluation units (EUs) in Nigeria

Figure S5. Average number of observations within a distance radius

Table S1. Data sources of the spatially referenced covariates

Table S2. Parameter estimates and 95% confidence intervals (CI) of the generalised linear model (defined as equation (1) in the manuscript) on the odds ratio (OR) scale for the lowest trachomatous inflammation—follicular (TF) prevalence evaluation units (EUs) in Ethiopia

Table S3. Parameter estimates and 95% confidence intervals (CI) of the generalised linear model (defined as equation (1) in the manuscript) on the odds ratio (OR) scale for the median trachomatous inflammation—follicular (TF) prevalence evaluation units (EUs) in Ethiopia

Table S4. Parameter estimates and 95% confidence intervals (CI) of the generalised linear model (defined as equation (1) in the manuscript) on the odds ratio (OR) scale for the highest trachomatous inflammation—follicular (TF) prevalence evaluation units (EUs) in Ethiopia

Table S5. Parameter estimates and 95% confidence intervals (CI) of the generalised linear model (defined as equation (1) in the manuscript) on the odds ratio (OR) scale for the lowest trachomatous inflammation—follicular (TF) prevalence evaluation units (EUs) in Malawi

Table S6. Parameter estimates and 95% confidence intervals (CI) of the generalised linear model (defined as equation (1) in the manuscript) on the odds ratio (OR) scale for the median trachomatous inflammation—follicular (TF) prevalence evaluation units (EUs) in Malawi

Table S7. Parameter estimates and 95% confidence intervals (CI) of the generalised linear model (defined as equation (1) in the manuscript) on the odds ratio (OR) scale for the highest trachomatous inflammation—follicular (TF) prevalence evaluation units (EUs) in Malawi

Table S8. Parameter estimates and 95% confidence intervals (CI) of the generalised linear model (defined as equation (1) in the manuscript) on the odds ratio (OR) scale for the lowest trachomatous inflammation—follicular (TF) prevalence evaluation units (EUs) in Niger

Table S9. Parameter estimates and 95% confidence intervals (CI) of the generalised linear model (defined as equation (1) in the manuscript) on the odds ratio (OR) scale for the median trachomatous inflammation—follicular (TF) prevalence evaluation units (EUs) in Niger

Table S10. Parameter estimates and 95% confidence intervals (CI) of the generalised linear model (defined as equation (1) in the manuscript) on the odds ratio (OR) scale for the highest trachomatous inflammation—follicular (TF) prevalence evaluation units (EUs) in Niger

Table S11. Parameter estimates and 95% confidence intervals (CI) of the generalised linear model (defined as equation (1) in the manuscript) on the odds ratio (OR) scale for the lowest trachomatous inflammation—follicular (TF) prevalence evaluation units (EUs) in Nigeria

Table S12. Parameter estimates and 95% confidence intervals (CI) of the generalised linear model (defined as equation (1) in the manuscript) on the odds ratio (OR) scale for the median trachomatous inflammation—follicular (TF) prevalence evaluation units (EUs) in Nigeria

Table S13. Parameter estimates and 95% confidence intervals (CI) of the generalised linear model (defined as equation (1) in the manuscript) on the odds ratio (OR) scale for the highest trachomatous inflammation—follicular (TF) prevalence evaluation units (EUs) in Nigeria


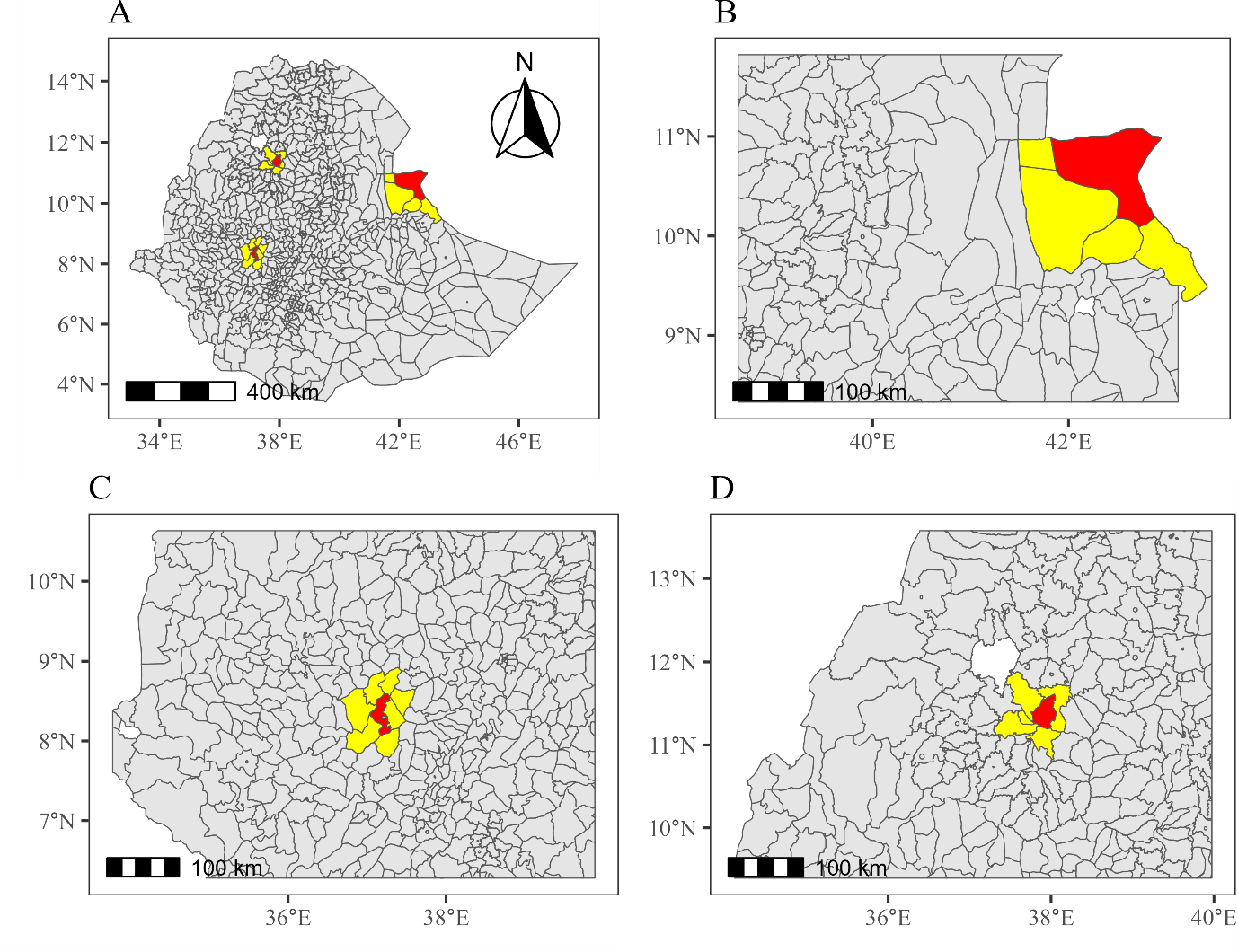


Figure S1. The analysed evaluation units (EUs) in Ethiopia (A). The red areas indicate the EUs that have the lowest (B), median (C), and highest (D) trachomatous inflammation—follicular (TF) prevalence. The yellow areas show the contiguous areas to the lowest, median, and highest EUs. Maps were created using shapefiles managed by the International Trachoma Initiative, with shapefile data sources including health ministries, OCHA, and the Humanitarian Data Exchange (<https://data.humdata.org/>). The boundaries and names shown and the designations used on this map do not imply the expression of any opinion whatsoever on the part of the authors, or the institutions with which they are affiliated, concerning the legal status of any country, territory, city or area or of its authorities, or concerning the delimitation of its frontiers or boundaries.


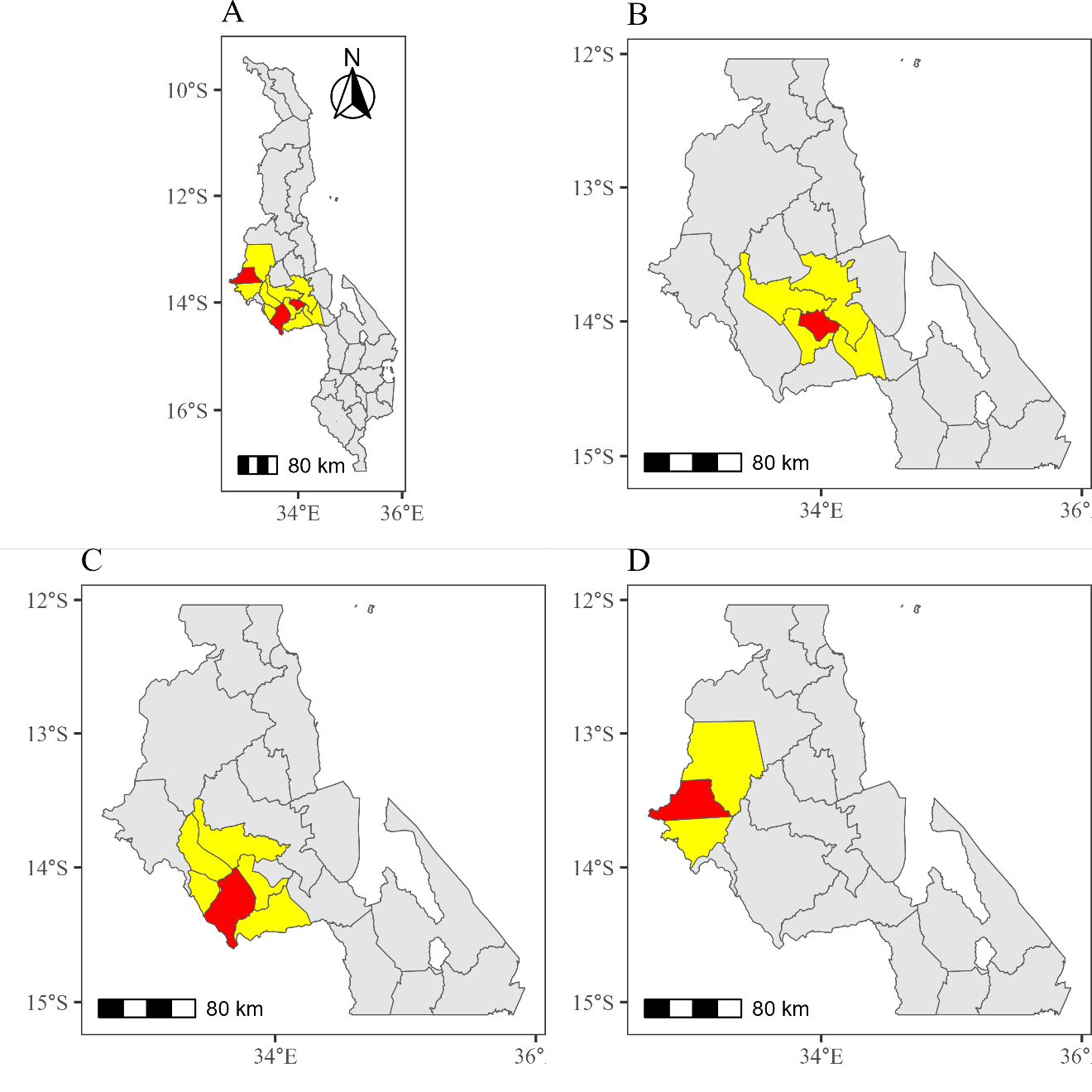


Figure S2. The analysed evaluation units (EUs) in Malawi (A). The red areas indicate the EUs that have the lowest (B), median (C), and highest (D) trachomatous inflammation—follicular (TF) prevalence. Maps were created using shapefiles managed by the International Trachoma Initiative, with shapefile data sources including health ministries, OCHA, and the Humanitarian Data Exchange (<https://data.humdata.org/>). The yellow areas show the contiguous areas to the lowest, median, and highest EUs. The boundaries and names shown and the designations used on this map do not imply the expression of any opinion whatsoever on the part of the authors, or the institutions with which they are affiliated, concerning the legal status of any country, territory, city or area or of its authorities, or concerning the delimitation of its frontiers or boundaries.


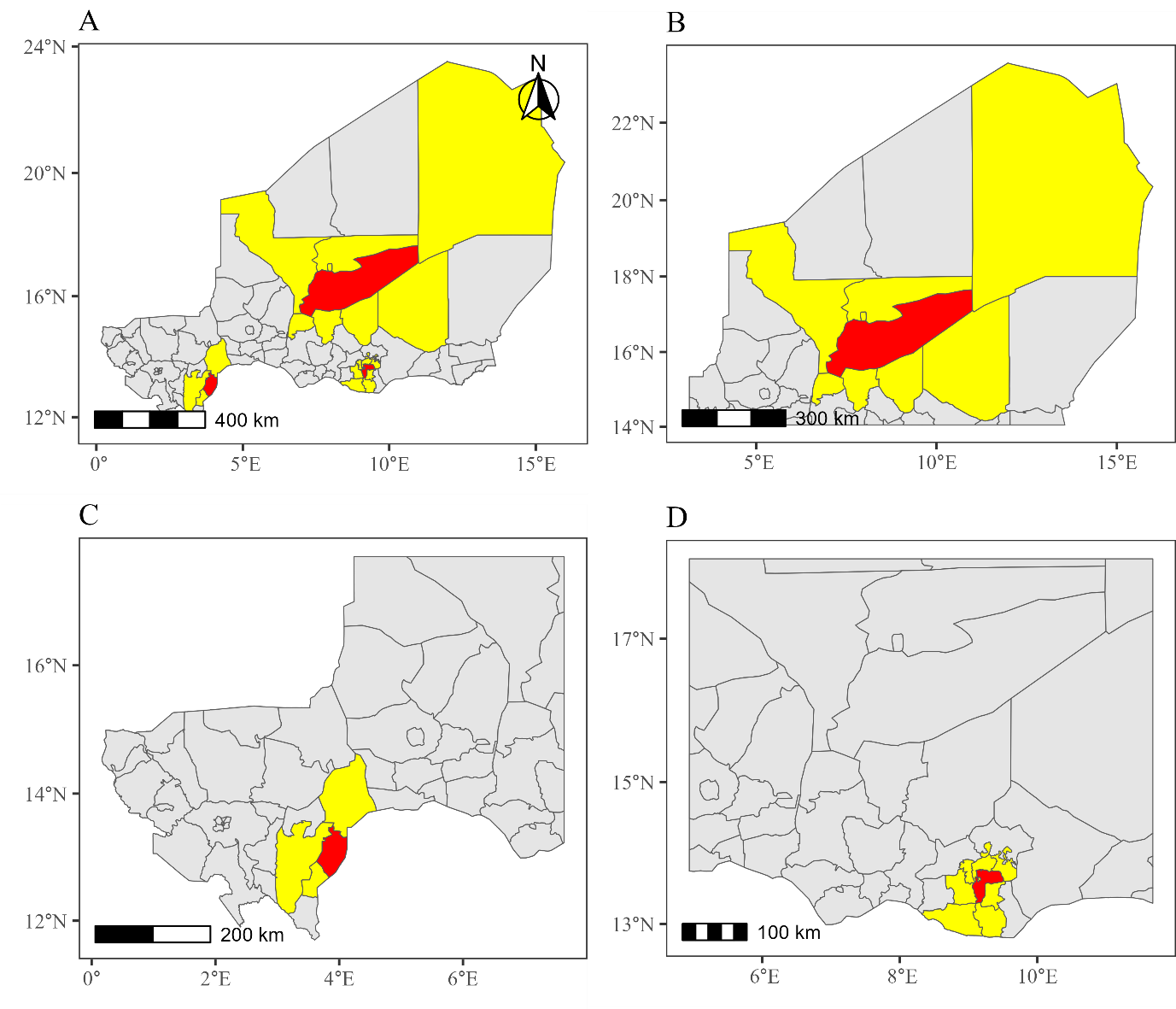


Figure S3. The analysed evaluation units (EUs) in Niger (A). The red areas indicate the EUs that have the lowest (B), median (C), and highest (D) trachomatous inflammation—follicular (TF) prevalence. The yellow areas show the contiguous areas to the lowest, median, and highest EUs. Maps were created using shapefiles managed by the International Trachoma Initiative, with shapefile data sources including health ministries, OCHA, and the Humanitarian Data Exchange (<https://data.humdata.org/>). The boundaries and names shown and the designations used on this map do not imply the expression of any opinion whatsoever on the part of the authors, or the institutions with which they are affiliated, concerning the legal status of any country, territory, city or area or of its authorities, or concerning the delimitation of its frontiers or boundaries.


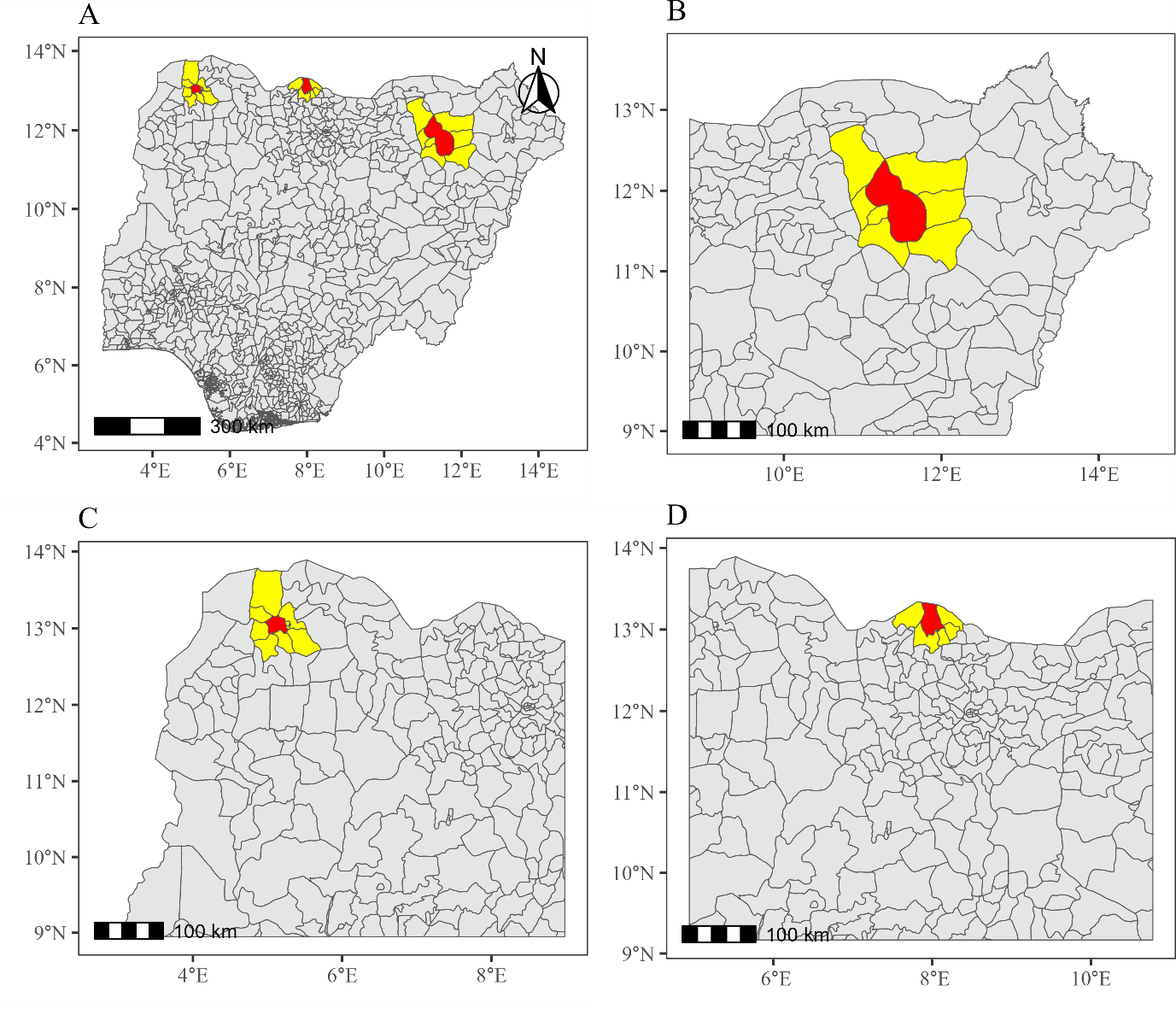


Figure S4. The analysed evaluation units (EUs) in Nigeria (A). The red areas indicate the EUs that have the lowest (B), median (C), and highest (D) trachomatous inflammation—follicular (TF) prevalence. The yellow areas show the contiguous areas to the lowest, median, and highest EUs. Maps were created using shapefiles managed by the International Trachoma Initiative, with shapefile data sources including health ministries, OCHA, and the Humanitarian Data Exchange (<https://data.humdata.org/>). The boundaries and names shown and the designations used on this map do not imply the expression of any opinion whatsoever on the part of the authors, or the institutions with which they are affiliated, concerning the legal status of any country, territory, city or area or of its authorities, or concerning the delimitation of its frontiers or boundaries.


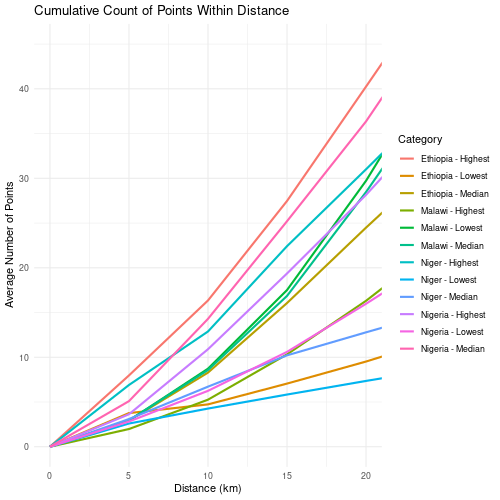


Figure S5. Average number of observations within a distance radius. Each curve corresponds to a specific country and TF prevalence level (Lowest, Median, Highest). The plot highlights spatial clustering patterns across datasets. Based on these curves, we calculate that within a radius of 5 km, the number of observations ranges from about 2 to 8 across data-sets; at 10 km, this range is from 4 to 16; and at 20 km, the number of average number of observations ranges between 7 and 40.

Table S1. Data sources of the spatially referenced covariates

| Category | Covariate | Source | Spatial resolution | Link | Reference |
| --- | --- | --- | --- | --- | --- |
| Environment | Precipitation | Climate Hazards Group InfraRed Precipitation with Station data (CHIRPS) | 0.05 degree (~5.55km) | Data: <https://data.chc.ucsb.edu/products/CHIRPS-2.0/global_annual/tifs/>  Description: <https://www.chc.ucsb.edu/data/chirps> | [1] |
|  | Temperature | Aqua Moderate Resolution Imaging Spectroradiometer (MODIS) Land Surface Temperature/Emissivity Monthly (MYD11C3) Version 6.1 product | 0.05 degree (~5.55km) | Data was obtained through R package ‘luna’  Description: <https://lpdaac.usgs.gov/products/myd11c3v061/> | [2, 3] |
|  | Enhanced vegetation index (EVI) | MODIS Vegetation Indices Monthly (MYD13A3) Version 6.1 product | 1 km | Data: <https://appeears.earthdatacloud.nasa.gov/>  Description: <https://lpdaac.usgs.gov/products/myd13a3v006/> | [4, 5] |
|  | Aridity index | Consultative Group on International Agricultural Research (CGIAR) Consortium for Spatial Information (CGIAR-CSI) | 30 arc seconds (~1 km) | Data and description: <https://figshare.com/articles/dataset/Global_Aridity_Index_and_Potential_Evapotranspiration_ET0_Climate_Database_v2/7504448/4> | [6, 7] |
|  | Altitude | WorldPop | 3 arc-second (~100m) | Data: <https://hub.worldpop.org/geodata/listing?id=58> | [8] |
| Accessibility | Travel time to cities | Malaria Atlas Project | 1 km | Data and description: <https://malariaatlas.org/project-resources/accessibility-to-healthcare/> | [9] |
|  | Travel time to healthcare (motorised and walking only) | Malaria Atlas Project | 1 km | Data and description: <https://malariaatlas.org/project-resources/accessibility-to-healthcare/> | [10] |
|  | Distance to OpenStreetMap (OSM) major roads | WorldPop | 3 arc-second (~100m) | Data: <https://hub.worldpop.org/geodata/listing?id=31> | [8, 11] |
|  | Distance to OSM major waterways | WorldPop | 3 arc-second (~100m) | Data: <https://hub.worldpop.org/geodata/listing?id=34> | [8, 11] |
| Water, sanitation and hygiene (WASH) | Percentage of population using an improved water source | DHS Program | 0.042 degree (~5 km) | Data and description: <https://spatialdata.dhsprogram.com/modeled-surfaces/#survey=AL\|2017\|DHS> | [12, 13] |
|  | Percentage of population using open defecation | DHS Program | 0.042 degree (~5 km) | Data and description: <https://spatialdata.dhsprogram.com/modeled-surfaces/#survey=AL\|2017\|DHS> | [12, 13] |
| Accessibility to and acceptance of health services | Percentage of children receiving at least one dose of diphtheria, tetanus toxoid, and pertussis (DPT) vaccine | DHS Program | 0.042 degree  (~5 km) | Data and description: <https://spatialdata.dhsprogram.com/modeled-surfaces/#survey=AL\|2017\|DHS> | [12, 13] |
|  | Percentage of children receiving Measles vaccination | DHS Program | 0.042 degree  (~5 km) | Data and description: <https://spatialdata.dhsprogram.com/modeled-surfaces/#survey=AL\|2017\|DHS> | [12, 13] |
|  | Percentage of live birth delivered at a health facility | DHS Program | 0.042 degree  (~5 km) | Data and description: <https://spatialdata.dhsprogram.com/modeled-surfaces/#survey=AL\|2017\|DHS> | [12, 13] |
|  | Proportion of population using insecticide-treated nets | Malaria Atlas Project | 1 km | Data and description: <https://malariaatlas.org/project-resources/modelling-coverage-of-insecticide-treated-nets-itns/> | [14, 15] |
| Ruralness | Nighttime lights | Earth Observation Group, Payne Institute for Public Policy (masked median radiance in V. 2.1 and 2.2 VNL Annual Product) | 15 arc seconds (~500m) | Data and description: <https://eogdata.mines.edu/products/vnl/> | [16–19] |
|  | Population density | WorldPop | 30 arc second (~1 km) | Data: <https://hub.worldpop.org/geodata/listing?id=77> | [8, 20] |
|  | Ruralness | European Commission Global Human Settlement Layer (GHSL) project | 1 km | Data and description: <https://ghsl.jrc.ec.europa.eu/download.php?ds=smod> | [21] |

Table S2. Parameter estimates and 95% confidence intervals (CI) of the generalised linear model (defined as Model 3 in the manuscript) on the odds ratio (OR) scale for the lowest trachomatous inflammation—follicular (TF) prevalence evaluation units (EUs) in Ethiopia

| Category | Variable | OR estimate (95% CI) |
| --- | --- | --- |
| Forced | Intercept | 0.01 (0.01, 0.02) |
|  | Age | 0.82 (0.69, 0.97) |
| Environment | Precipitation | 0.50 (0.35, 0.74) |
|  | Aridity index | 4.17 (3.10, 5.63) |
| Accessibility | Log travel time to cities | 1.51 (1.11, 2.04) |
|  | Log travel time to healthcare (motorised) | 0.73 (0.55, 0.97) |
| Accessibility to and acceptance of health services | Percentage of children receiving Measles vaccination | 0.82 (0.65, 1.03) |

Table S3. Parameter estimates and 95% confidence intervals (CI) of the generalised linear model (defined as Model 3 in the manuscript) on the odds ratio (OR) scale for the median trachomatous inflammation—follicular (TF) prevalence evaluation units (EUs) in Ethiopia

| Category | Variable | OR estimate (95% CI) |
| --- | --- | --- |
| Forced | Intercept | 0.16 (0.10, 0.25) |
|  | Age | 1.69 (1.12, 2.57) |
|  | $\max\left( Age-3, 0 \right)$ | 0.25 (0.15, 0.41) |
| Environment | Precipitation | 2.43 (1.40, 4.20) |
|  | EVI | 1.57 (1.22, 2.03) |
|  | Aridity index | 0.32 (0.16, 0.66) |
| Accessibility | Travel time to cities | 0.76 (0.66, 0.88) |
|  | Travel time to healthcare (motorised) | 1.41 (1.28, 1.55) |
| WASH | Percentage of population using an improved water source | 1.15 (0.97, 1.37) |
|  | Percentage of population using open defecation | 1.22 (1.03, 1.45) |
| Accessibility to and acceptance of health services | Percentage of children receiving Measles vaccination | 0.58 (0.49, 0.69) |
| Ruralness | Nighttime lights | 1.15 (1.01, 1.30) |

Table S4. Parameter estimates and 95% confidence intervals (CI) of the generalised linear model (defined as Model 3 in the manuscript) on the odds ratio (OR) scale for the highest trachomatous inflammation—follicular (TF) prevalence evaluation units (EUs) in Ethiopia

| Category | Variable | OR estimate (95% CI) |
| --- | --- | --- |
| Forced | Intercept | 2.84 (1.29, 6.29) |
|  | Age | 4.26 (2.22, 8.18) |
|  | $\max\left( Age-2, 0 \right)$ | 0.10 (0.05, 0.20) |
| Environment | Precipitation | 1.46 (1.14, 1.88) |
|  | Temperature | 1.47 (1.04, 2.10) |
|  | EVI | 0.75 (0.64, 0.87) |
|  | Aridity index | 0.45 (0.33, 0.61) |
|  | Altitude | 1.52 (1.15, 2.02) |
| Accessibility | Travel time to healthcare (walking only) | 0.86 (0.76, 0.96) |
|  | Log distance to OSM major roads | 1.77 (1.52, 2.06) |
|  | Distance to OSM major waterways | 1.25 (1.09, 1.44) |
| WASH | Percentage of population using an improved water source | 1.64 (1.35, 2.00) |
|  | Percentage of population using open defecation | 1.63 (1.20, 2.21) |
| Accessibility to and acceptance of health services | Percentage of children receiving at least one dose of DPT vaccine | 0.22 (0.15, 0.31) |
|  | Percentage of children receiving Measles vaccination | 2.30 (1.85, 2.86) |
|  | Percentage of live birth delivered at a health facility | 2.18 (1.56, 3.05) |
| Ruralness | Nighttime lights | 0.41 (0.29, 0.58) |
|  | Population density | 1.93 (1.51, 2.46) |
|  | Rural (reference) | - |
|  | Urban | 0.09 (0.04, 0.23) |

Table S5. Parameter estimates and 95% confidence intervals (CI) of the generalised linear model (defined as Model 3 in the manuscript) on the odds ratio (OR) scale for the lowest trachomatous inflammation—follicular (TF) prevalence evaluation units (EUs) in Malawi

| Category | Variable | OR estimate (95% CI) |
| --- | --- | --- |
| Forced | Intercept | 0.02 (0.01, 0.04) |
|  | Age | 1.45 (0.70, 3.00) |
|  | $\max\left( Age-4, 0 \right)$ | 0.51 (0.19, 1.39) |
| Environment | EVI | 0.76 (0.59, 0.99) |
| Accessibility | Travel time to cities | 1.29 (1.03, 1.61) |

Table S6. Parameter estimates and 95% confidence intervals (CI) of the generalised linear model (defined as Model 3 in the manuscript) on the odds ratio (OR) scale for the median trachomatous inflammation—follicular (TF) prevalence evaluation units (EUs) in Malawi

| Category | Variable | OR estimate (95% CI) |
| --- | --- | --- |
| Forced | Intercept | 0.03 (0.01, 0.08) |
|  | Age | 1.63 (0.65, 4.06) |
|  | $\max\left( Age-3, 0 \right)$ | 0.36 (0.12, 1.09) |
| Accessibility | Travel time to cities | 1.23 (0.98, 1.56) |
|  | Travel time to healthcare (motorised) | 0.55 (0.33, 0.91) |
|  | Travel time to healthcare (walking only) | 1.87 (1.19, 2.93) |
| WASH | Percentage of population using open defecation | 0.85 (0.67, 1.07) |
| Ruralness | Log nighttime lights | 0.85 (0.64, 1.14) |

Table S7. Parameter estimates and 95% confidence intervals (CI) of the generalised linear model (defined as Model 3 in the manuscript) on the odds ratio (OR) scale for the highest trachomatous inflammation—follicular (TF) prevalence evaluation units (EUs) in Malawi

| Category | Variable | OR estimate (95% CI) |
| --- | --- | --- |
| Forced | Intercept | 0.05 (0.03, 0.09) |
|  | Age | 1.62 (0.90, 2.90) |
|  | $\max\left( Age-4, 0 \right)$ | 0.22 (0.09, 0.53) |
| Environment | Temperature | 0.71 (0.45, 1.11) |
|  | Altitude | 0.65 (0.37, 1.14) |
| Accessibility | Travel time to cities | 2.20 (1.59, 3.04) |
|  | Log travel time to healthcare (motorised) | 0.63 (0.36, 1.09) |
|  | Travel time to healthcare (walking only) | 1.39 (0.92, 2.10) |
|  | Distance to OSM major roads | 0.75 (0.57, 0.98) |
|  | Distance to OSM major waterways | 1.31 (0.97, 1.77) |

Table S8. Parameter estimates and 95% confidence intervals (CI) of the generalised linear model (defined as Model 3 in the manuscript) on the odds ratio (OR) scale for the lowest trachomatous inflammation—follicular (TF) prevalence evaluation units (EUs) in Niger

| Category | Variable | OR estimate (95% CI) |
| --- | --- | --- |
| Forced | Intercept | 0.07 (0.04, 0.13) |
|  | Age | 2.89 (1.68, 4.95) |
|  | $\max\left( Age-3, 0 \right)$ | 0.15 (0.08, 0.28) |
| Environment | Precipitation | 1.90 (1.4, 2.58) |
|  | Temperature | 0.39 (0.31, 0.50) |
|  | Aridity index | 0.42 (0.31, 0.58) |
| Accessibility | Log travel time to healthcare (motorised) | 2.94 (2.11, 4.10) |
|  | Log travel time to healthcare (walking only) | 0.32 (0.23, 0.44) |
|  | Log distance to OSM major roads | 0.50 (0.40, 0.62) |
|  | Log distance to OSM major waterways | 0.64 (0.52, 0.79) |
| Accessibility to and acceptance of health services | Proportion of population using insecticide-treated nets | 0.57 (0.40, 0.81) |
| Ruralness | Log population density | 0.58 (0.45, 0.74) |
|  | Rural (reference) | - |
|  | Urban | 3.03 (1.33, 6.91) |

Table S9. Parameter estimates and 95% confidence intervals (CI) of the generalised linear model (defined as Model 3 in the manuscript) on the odds ratio (OR) scale for the median trachomatous inflammation—follicular (TF) prevalence evaluation units (EUs) in Niger

| Category | Variable | OR estimate (95% CI) |
| --- | --- | --- |
| Forced | Intercept | 0.05 (0.03, 0.08) |
|  | Age | 2.20 (1.26, 3.84) |
|  | $\max\left( Age-4, 0 \right)$ | 0.09 (0.04, 0.21) |
| Environment | Precipitation | 0.08 (0.03, 0.24) |
|  | Aridity index | 11.3 (3.59, 35.53) |
|  | Altitude | 0.31 (0.18, 0.52) |
| Accessibility | Log travel time to healthcare (motorised) | 1.36 (0.91, 2.05) |
|  | Log travel time to healthcare (walking only) | 0.59 (0.41, 0.86) |
|  | Log distance to OSM major roads | 1.57 (1.19, 2.07) |
| Accessibility to and acceptance of health services | Proportion of population using insecticide-treated nets | 0.56 (0.41, 0.78) |
| Ruralness | Nighttime lights | 1.24 (0.95, 1.61) |

Table S10. Parameter estimates and 95% confidence intervals (CI) of the generalised linear model (defined as Model 3 in the manuscript) on the odds ratio (OR) scale for the highest trachomatous inflammation—follicular (TF) prevalence evaluation units (EUs) in Niger

| Category | Variable | OR estimate (95% CI) |
| --- | --- | --- |
| Forced | Intercept | 0.17 (0.12, 0.26) |
|  | Age | 2.96 (2.05, 4.27) |
|  | $\max\left( Age-3, 0 \right)$ | 0.13 (0.08, 0.20) |
| Environment | Precipitation | 1.38 (1.15, 1.66) |
|  | Temperature | 1.13 (1.01, 1.26) |
|  | EVI | 1.13 (0.99, 1.30) |
|  | Aridity index | 0.54 (0.42, 0.70) |
| Accessibility | Log travel time to cities | 1.31 (1.04, 1.66) |
|  | Log travel time to healthcare (motorised) | 0.68 (0.55, 0.84) |
|  | Log travel time to healthcare (walking only) | 1.27 (1.11, 1.45) |
|  | Distance to OSM major waterways | 0.87 (0.77, 1.00) |
| Accessibility to and acceptance of health services | Proportion of population using insecticide-treated nets | 0.88 (0.79, 0.98) |
| Ruralness | Nighttime lights | 0.75 (0.60, 0.94) |
|  | Log population density | 0.95 (0.77, 1.17) |

Table S11. Parameter estimates and 95% confidence intervals (CI) of the generalised linear model (defined as Model 3 in the manuscript) on the odds ratio (OR) scale for the lowest trachomatous inflammation—follicular (TF) prevalence evaluation units (EUs) in Nigeria

| Category | Variable | OR estimate (95% CI) |
| --- | --- | --- |
| Forced | Intercept | 0.06 (0.03, 0.11) |
|  | Age | 2.40 (1.41, 4.11) |
|  | $\max\left( Age-3, 0 \right)$ | 0.17 (0.09, 0.32) |
| Environment | Precipitation | 0.63 (0.51, 0.78) |
| Accessibility | Log travel time to cities | 0.49 (0.36, 0.67) |
|  | Travel time to healthcare (walking only) | 1.20 (1.04, 1.40) |
|  | Log distance to OSM major roads | 1.38 (1.10, 1.73) |
|  | Distance to OSM major waterways | 1.62 (1.36, 1.93) |
| Accessibility to and acceptance of health services | Percentage of children receiving at least one dose of DPT vaccine | 0.78 (0.58, 1.04) |
|  | Percentage of live birth delivered at a health facility | 1.56 (1.23, 1.98) |
| Ruralness | Log nighttime lights | 1.53 (1.24, 1.88) |
|  | Log population density | 0.33 (0.26, 0.42) |

Table S12. Parameter estimates and 95% confidence intervals (CI) of the generalised linear model (defined as Model 3 in the manuscript) on the odds ratio (OR) scale for the median trachomatous inflammation—follicular (TF) prevalence evaluation units (EUs) in Nigeria

| Category | Variable | OR estimate (95% CI) |
| --- | --- | --- |
| Forced | Intercept | 0.06 (0.03, 0.12) |
|  | Age | 1.86 (1.02, 3.38) |
|  | $\max\left( Age-3, 0 \right)$ | 0.22 (0.11, 0.45) |
| Environment | Precipitation | 1.97 (1.27, 3.05) |
|  | Aridity index | 0.53 (0.35, 0.80) |
|  | Altitude | 0.55 (0.46, 0.65) |
| Accessibility | Travel time to cities | 1.37 (1.03, 1.82) |
|  | Travel time to healthcare (walking only) | 0.81 (0.65, 1.00) |
|  | Distance to OSM major waterways | 0.65 (0.47, 0.89) |
| Accessibility to and acceptance of health services | Percentage of children receiving at least one dose of DPT vaccine | 0.32 (0.11, 0.93) |
|  | Percentage of children receiving Measles vaccination | 1.92 (0.87, 4.28) |
|  | Log percentage of live birth delivered at a health facility | 2.12 (1.22, 3.69) |
| Ruralness | Log nighttime lights | 0.37 (0.25, 0.56) |

Table S13. Parameter estimates and 95% confidence intervals (CI) of the generalised linear model (defined as Model 3 in the manuscript) on the odds ratio (OR) scale for the highest trachomatous inflammation—follicular (TF) prevalence evaluation units (EUs) in Nigeria

| Category | Variable | OR estimate (95% CI) |
| --- | --- | --- |
| Forced | Intercept | 0.03 (0.02, 0.05) |
|  | Age | 2.88 (1.85, 4.48) |
|  | $\max\left( Age-4, 0 \right)$ | 0.30 (0.17, 0.53) |
| Environment | Precipitation | 0.58 (0.41, 0.82) |
|  | Temperature | 0.72 (0.45, 1.15) |
|  | Aridity index | 0.14 (0.07, 0.30) |
|  | Altitude | 0.70 (0.51, 0.96) |
| Accessibility | Travel time to cities | 0.18 (0.1, 0.33) |
|  | Travel time to healthcare (motorised) | 1.75 (1.26, 2.43) |
|  | Distance to OSM major roads | 1.34 (1.09, 1.65) |
| WASH | Percentage of population using open defecation | 0.58 (0.41, 0.83) |
| Accessibility to and acceptance of health services | Percentage of children receiving Measles vaccination | 1.39 (1.03, 1.87) |
|  | Percentage of live birth delivered at a health facility | 0.37 (0.29, 0.48) |
|  | Proportion of population using insecticide-treated nets | 2.12 (1.50, 2.99) |
| Ruralness | Log nighttime lights | 0.78 (0.58, 1.07) |
|  | Log population density | 0.34 (0.22, 0.51) |

References

1. Funk C, Peterson P, Landsfeld M, Pedreros D, Verdin J, Shukla S, et al. The climate hazards infrared precipitation with stations - A new environmental record for monitoring extremes. Sci Data. 2015;2.

2. Wan Z. MODIS Land-Surface Temperature Algorithm Theoretical Basis Document (LST ATBD) Version 3.3. Santa Barbara, California, USA; 1999.

3. Wan Z. Collection-6 MODIS Land Surface Temperature Products Users’ Guide. Santa Barbara, California, USA; 2013.

4. Huete A, Justice C, Leeuwen W van. MODIS  Vegetation Index (MOD 13) Algorithm Theoretical Basis Document Version 3. 1999.

5. Didan K, Munoz AB, Solano R, Huete A. MODIS Vegetation Index User’s Guide (MOD13 Series) Version 3.00. Tucson, Arizona, USA; 2015.

6. Zomer RJ, Trabucco A, Straaten O van, Bossio DA. Carbon, Land and Water: A Global Analysis of the Hydrologic Dimensions of Climate Change Mitigation through Afforestation/Reforestation. Colombo, Sri Lanka; 2007.

7. Zomer RJ, Trabucco A, Bossio DA, Verchot L V. Climate change mitigation: A spatial analysis of global land suitability for clean development mechanism afforestation and reforestation. Agric Ecosyst Environ. 2008;126.

8. Lloyd CT, Chamberlain H, Kerr D, Yetman G, Pistolesi L, Stevens FR, et al. Global spatio-temporally harmonised datasets for producing high-resolution gridded population distribution datasets. Big Earth Data. 2019;3.

9. Weiss DJ, Nelson A, Gibson HS, Temperley W, Peedell S, Lieber A, et al. A global map of travel time to cities to assess inequalities in accessibility in 2015. Nature. 2018;553.

10. Weiss DJ, Nelson A, Vargas-Ruiz CA, Gligorić K, Bavadekar S, Gabrilovich E, et al. Global maps of travel time to healthcare facilities. Nat Med. 2020;26.

11. Lloyd CT. High resolution global gridded data for use in population studies. In: International Archives of the Photogrammetry, Remote Sensing and Spatial Information Sciences - ISPRS Archives. 2017.

12. Gething P, Tatem A, Bird T, Burgert-Brucker CR. Creating Spatial Interpolation Surfaces with DHS Data DHS Spatial Analysis Reports No. 11. Rockville, Maryland, USA; 2015.

13. Burgert-Brucker CR, Dontamsetti T, Marshall AMJ, Gething PW. Guidance for Use of The DHS Program Modeled Map Surfaces. DHS Spatial Analysis Reports No. 14. Rockville, Maryland, USA; 2016.

14. Bertozzi-Villa A, Bever CA, Koenker H, Weiss DJ, Vargas-Ruiz C, Nandi AK, et al. Maps and metrics of insecticide-treated net access, use, and nets-per-capita in Africa from 2000-2020. Nat Commun. 2021;12.

15. Bhatt S, Weiss DJ, Mappin B, Dalrymple U, Cameron E, Bisanzio D, et al. Coverage and system efficiencies of insecticide-treated nets in Africa from 2000 to 2017. Elife. 2015;4.

16. Elvidge CD, Hsu FC, Zhizhin M, Ghosh T, Taneja J, Bazilian M. Indicators of electric power instability from satellite observed nighttime lights. Remote Sens (Basel). 2020;12.

17. Elvidge CD, Baugh KE, Zhizhin M, Hsu F-C. Why VIIRS data are superior to DMSP for mapping nighttime lights. Proceedings of the Asia-Pacific Advanced Network. 2013;35.

18. Elvidge CD, Baugh K, Zhizhin M, Hsu FC, Ghosh T. VIIRS night-time lights. Int J Remote Sens. 2017;38.

19. Elvidge CD, Zhizhin M, Ghosh T, Hsu FC, Taneja J. Annual time series of global viirs nighttime lights derived from monthly averages: 2012 to 2019. Remote Sens (Basel). 2021;13.

20. Stevens FR, Gaughan AE, Linard C, Tatem AJ. Disaggregating census data for population mapping using Random forests with remotely-sensed and ancillary data. PLoS One. 2015;10.

21. European Commission. GHSL Data Package 2023. Luxembourg; 2023.
