# Supplementary material for: Understanding the impact of covariates for trachoma prevalence prediction using geostatistical methods
Source: BMC Glob Public Health. 2025 Jun 1;3:48. doi: 10.1186/s44263-025-00161-x (PMC12126867; doi:10.1186/s44263-025-00161-x)
Supplement: Supplementary file 2 — Additional file 2: Provides information on the handling of spatially referenced covariates used in the geostatistical models. Figure S6. Annual average land surface temperature (LST) for the year 2016–22 in Ethiopia. Figure S7. Annual average land surface temperature (LST) for the year 2016–22 in Malawi. Figure S8. Annual average land surface temperature (LST) for the year 2016–22 in Niger. Figure S9. Annual average land surface temperature (LST) for the year 2016–22 in Nigeria. Figure S10. Observed temperature (in Kelvin) and fitted values at randomly selected pixels that contained missing values in Ethiopia . Figure S11. Observed temperature (in Kelvin) and fitted values at randomly selected pixels that contained missing values in Malawi . Figure S12. Observed temperature (in Kelvin) and fitted values at randomly selected pixels that contained missing values in Niger. Figure S13. Observed temperature (in Kelvin) and fitted values at randomly selected pixels that contained missing values in Nigeria. [file 44263_2025_161_MOESM2_ESM.docx]

**Understanding the impact of covariates for trachoma prevalence prediction using geostatistical methods**

Additional file 2

Figure S6. Annual average land surface temperature (LST) for the year 2016–22 in Ethiopia

Figure S7. Annual average land surface temperature (LST) for the year 2016–22 in Malawi

Figure S8. Annual average land surface temperature (LST) for the year 2016–22 in Niger

Figure S9. Annual average land surface temperature (LST) for the year 2016–22 in Nigeria

Figure S10. Observed temperature (in Kelvin) and fitted values at randomly selected pixels that contained missing values in Ethiopia

Figure S11. Observed temperature (in Kelvin) and fitted values at randomly selected pixels that contained missing values in Malawi

Figure S12. Observed temperature (in Kelvin) and fitted values at randomly selected pixels that contained missing values in Niger

Figure S13. Observed temperature (in Kelvin) and fitted values at randomly selected pixels that contained missing values in Nigeria

**The time series model for temperature values**

We describe the statistical model that was used to interpolate the missing values in temperature data.

We used the Aqua Moderate Resolution Imaging Spectroradiometer (MODIS) Land Surface Temperature/Emissivity Monthly (MYD11C3) Version 6.1 product for temperature from the year 2016 to 2022, obtained through the ‘luna’ package in R. The data had some missing values as shown in Figure S1–4. The data were created by satellite remote sensing, and cloud cover is often the source of missing or low-quality data [1].

We interpolated the missing values based on the time series model which we define as follows.

$$y=\alpha+ \beta_{1}\sin\left( 2*\pi*\frac{t}{12} \right)+\beta_{2}\cos\left( 2*\pi*\frac{t}{12} \right)+\beta_{3}\sin\left( 2*\pi*\frac{t}{6} \right)+\beta_{4}\cos\left( 2*\pi*\frac{t}{6} \right)+S\left( t \right)+Z(t)$$

where $y$ is temperature, and $\alpha$ and $\beta$, respectively, represent the intercept and regression coefficients. $t$ is a monthly time. $S(t)$ is a Matern process with variance $\sigma^{2}$, scale parameter $\varphi$, and smoothness $\kappa= 0.5$, and $Z(t)$ is Gaussian noise with variance $\tau^{2}$.

For each pixel that had a missing value, we fitted the model to the observed values. The parameters were estimated by maximum likelihood using an R package ‘PrevMap’. Figure S5–8 shows the predicted values compared to observed values at a randomly selected pixel for each country.


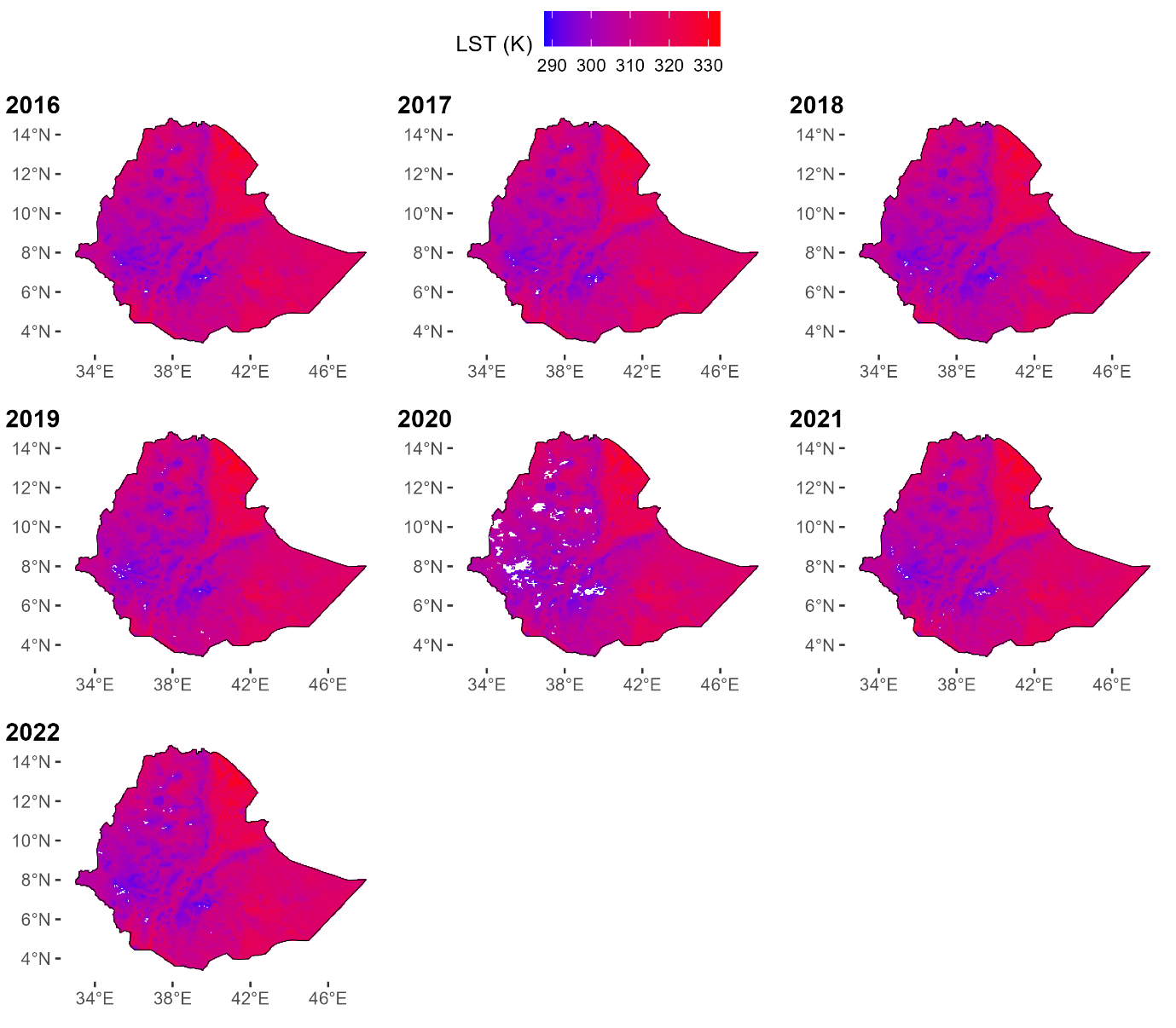


Figure S6. Annual average land surface temperature (LST) for the year 2016–22 in Ethiopia. The annual average was calculated by taking the mean of the original monthly data. The white areas show the pixels that have missing values at least for a month. Maps were created using shapefiles managed by the International Trachoma Initiative, with shapefile data sources including health ministries, OCHA, and the Humanitarian Data Exchange (<https://data.humdata.org/>). The boundaries and names shown and the designations used on this map do not imply the expression of any opinion whatsoever on the part of the authors, or the institutions with which they are affiliated, concerning the legal status of any country, territory, city or area or of its authorities, or concerning the delimitation of its frontiers or boundaries.


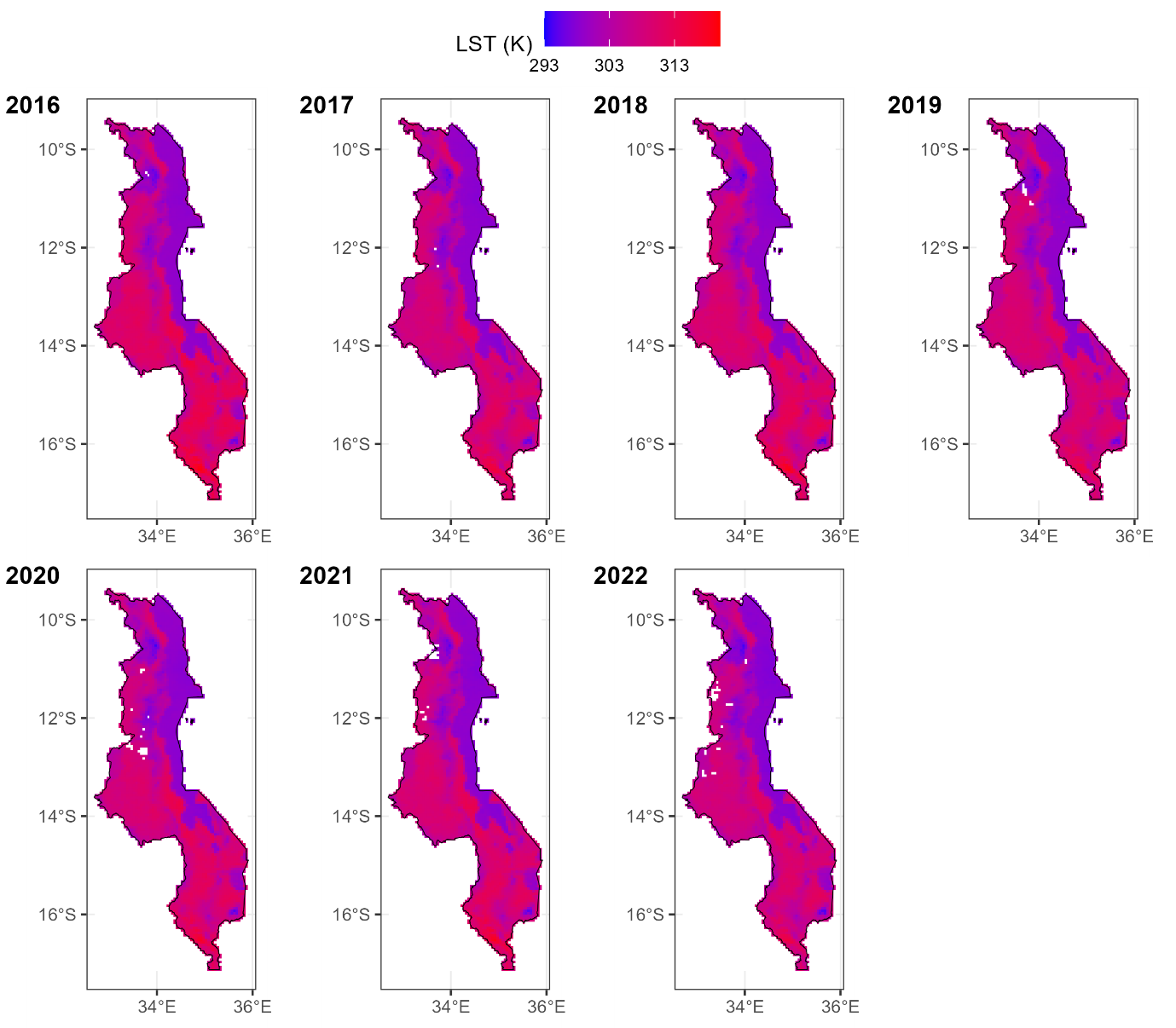


Figure S7. Annual average land surface temperature (LST) for the year 2016–22 in Malawi. The annual average was calculated by taking the mean of the original monthly data. The white areas show the pixels that have missing values at least for a month. Maps were created using shapefiles managed by the International Trachoma Initiative, with shapefile data sources including health ministries, OCHA, and the Humanitarian Data Exchange (<https://data.humdata.org/>). The boundaries and names shown and the designations used on this map do not imply the expression of any opinion whatsoever on the part of the authors, or the institutions with which they are affiliated, concerning the legal status of any country, territory, city or area or of its authorities, or concerning the delimitation of its frontiers or boundaries.


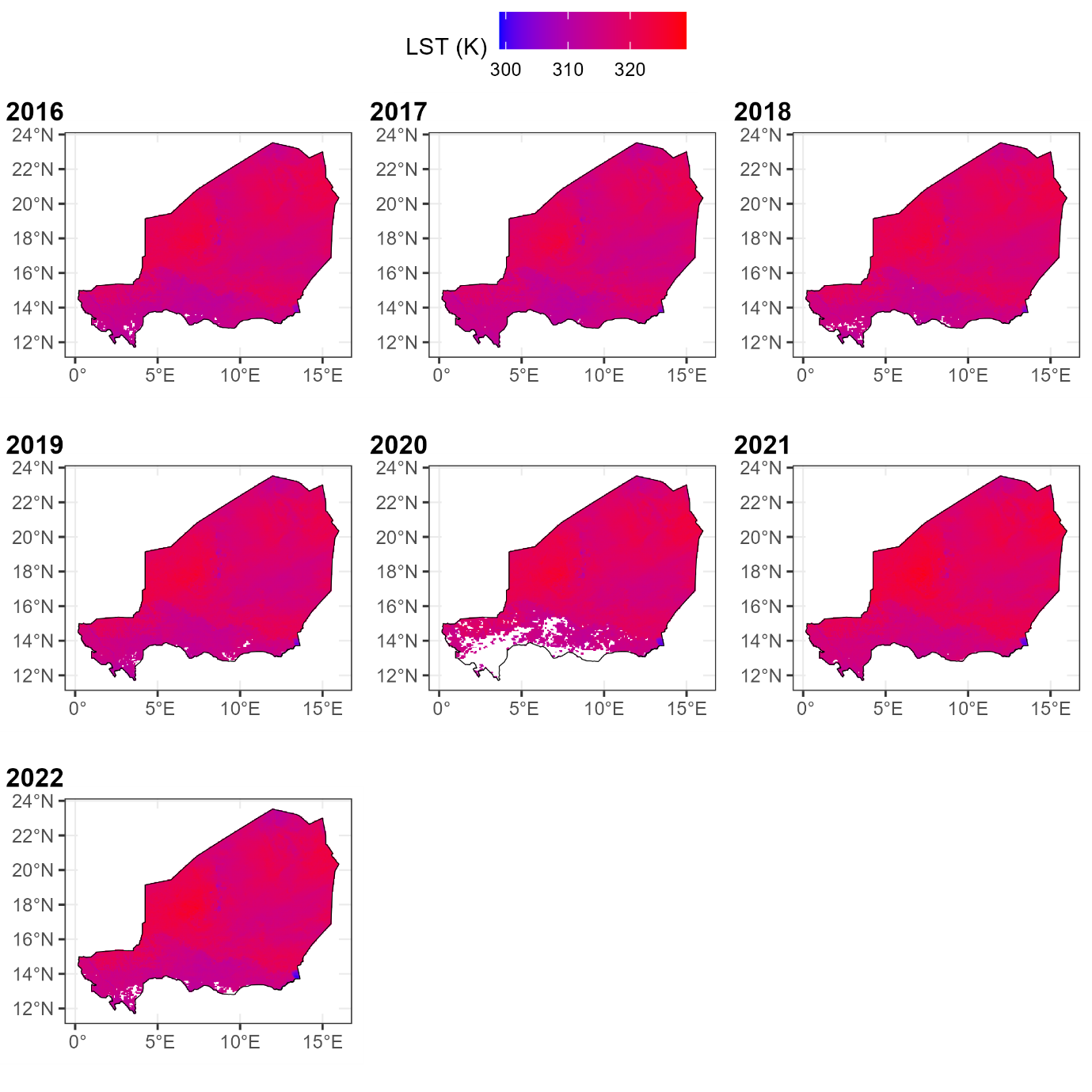


Figure S8. Annual average land surface temperature (LST) for the year 2016–22 in Niger. The annual average was calculated by taking the mean of the original monthly data. The white areas show the pixels that have missing values at least for a month. Maps were created using shapefiles managed by the International Trachoma Initiative, with shapefile data sources including health ministries, OCHA, and the Humanitarian Data Exchange (<https://data.humdata.org/>). The boundaries and names shown and the designations used on this map do not imply the expression of any opinion whatsoever on the part of the authors, or the institutions with which they are affiliated, concerning the legal status of any country, territory, city or area or of its authorities, or concerning the delimitation of its frontiers or boundaries.


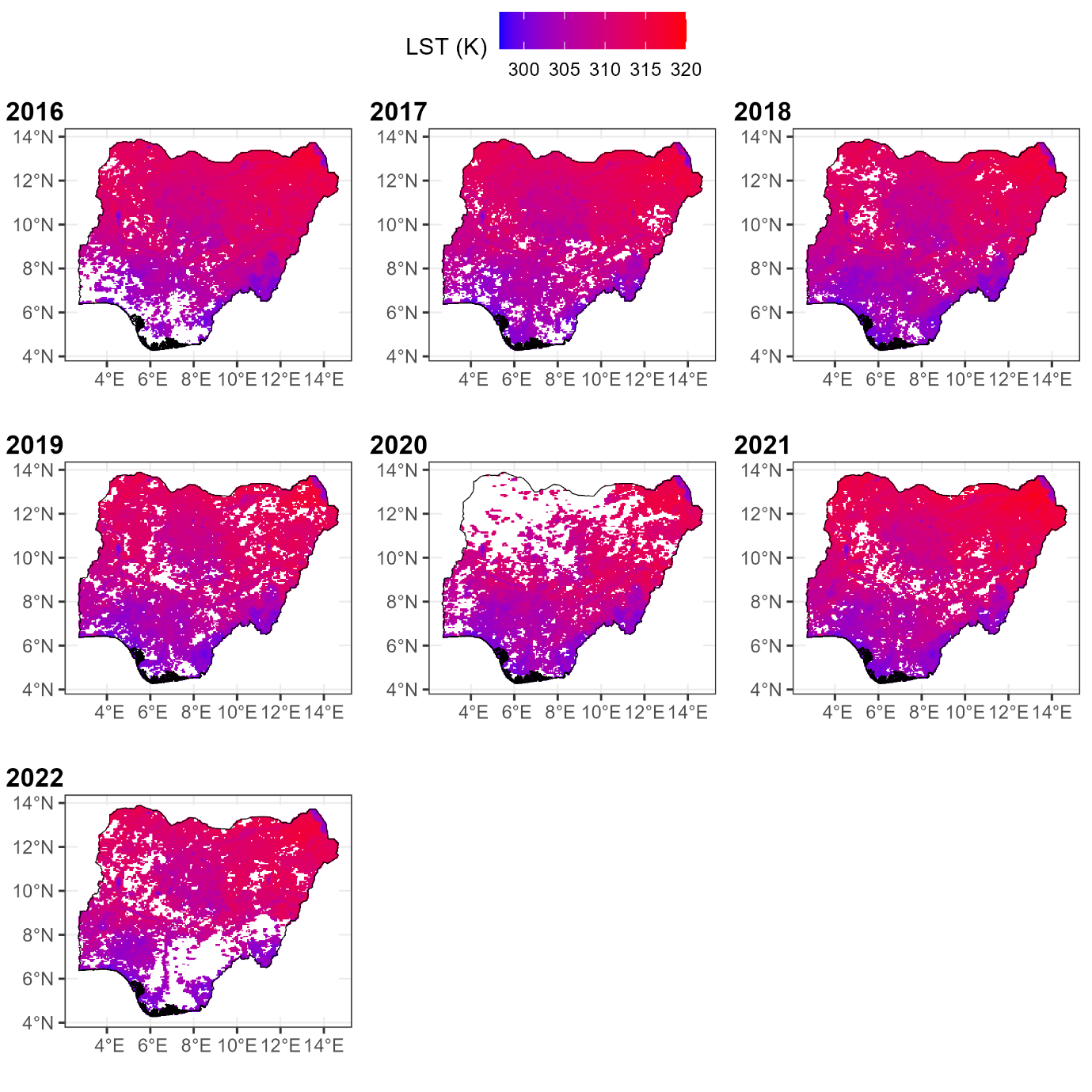


Figure S9. Annual average land surface temperature (LST) for the year 2016–22 in Nigeria. The annual average was calculated by taking the mean of the original monthly data. The white areas show the pixels that have missing values at least for a month. Maps were created using shapefiles managed by the International Trachoma Initiative, with shapefile data sources including health ministries, OCHA, and the Humanitarian Data Exchange (<https://data.humdata.org/>). The boundaries and names shown and the designations used on this map do not imply the expression of any opinion whatsoever on the part of the authors, or the institutions with which they are affiliated, concerning the legal status of any country, territory, city or area or of its authorities, or concerning the delimitation of its frontiers or boundaries.


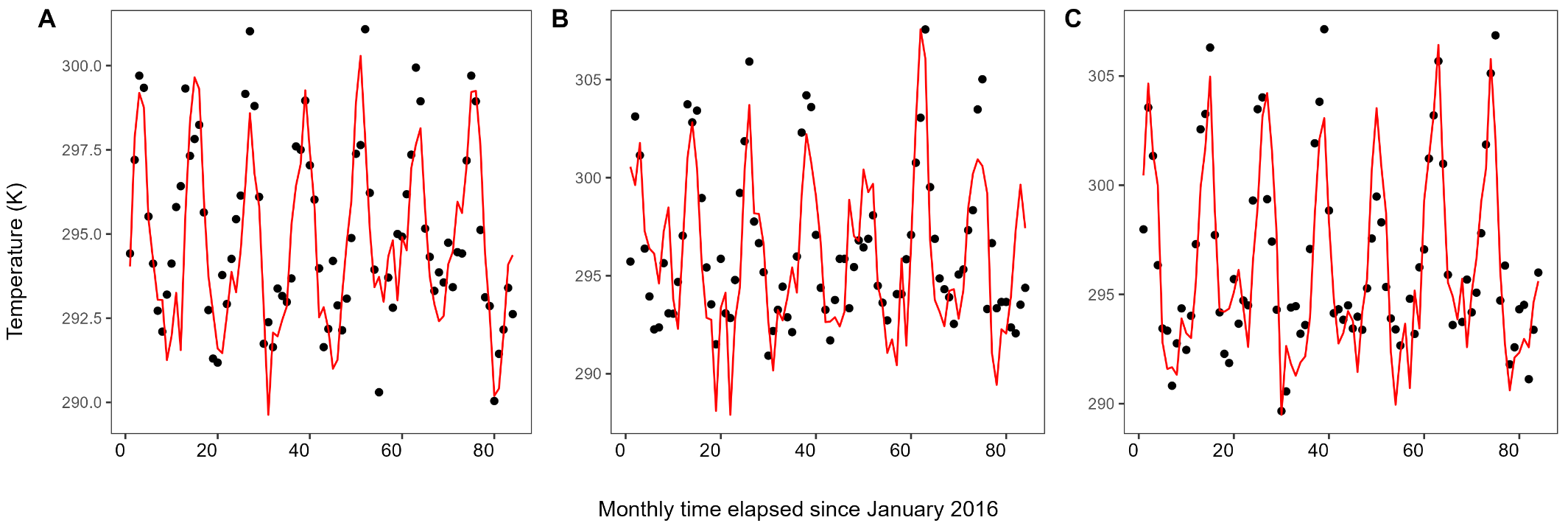


Figure S10. Observed temperature (in Kelvin) and fitted values at randomly selected pixels that contained missing values in Ethiopia. The black dots are observed temperature values, and the red line shows the predicted values. The pixels were selected randomly among the ones that had at least one missing value.


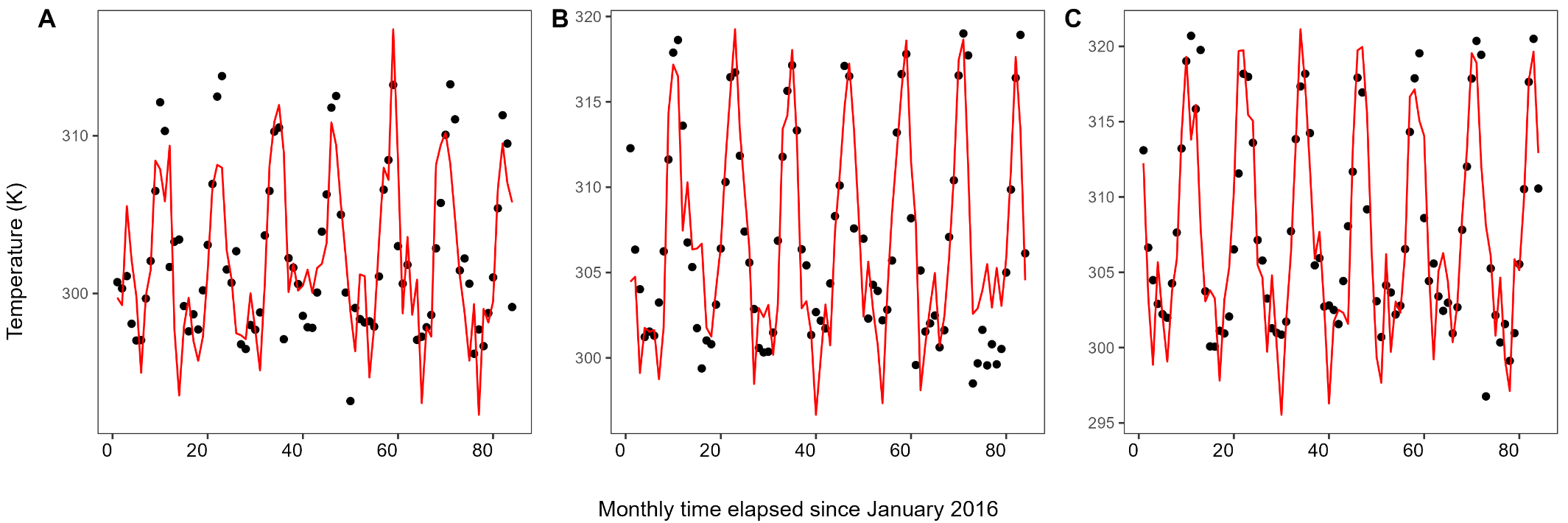


Figure S11. Observed temperature (in Kelvin) and fitted values at randomly selected pixels that contained missing values in Malawi. The black dots are observed temperature values, and the red line shows the predicted values. The pixels were selected randomly among the ones that had at least one missing value.


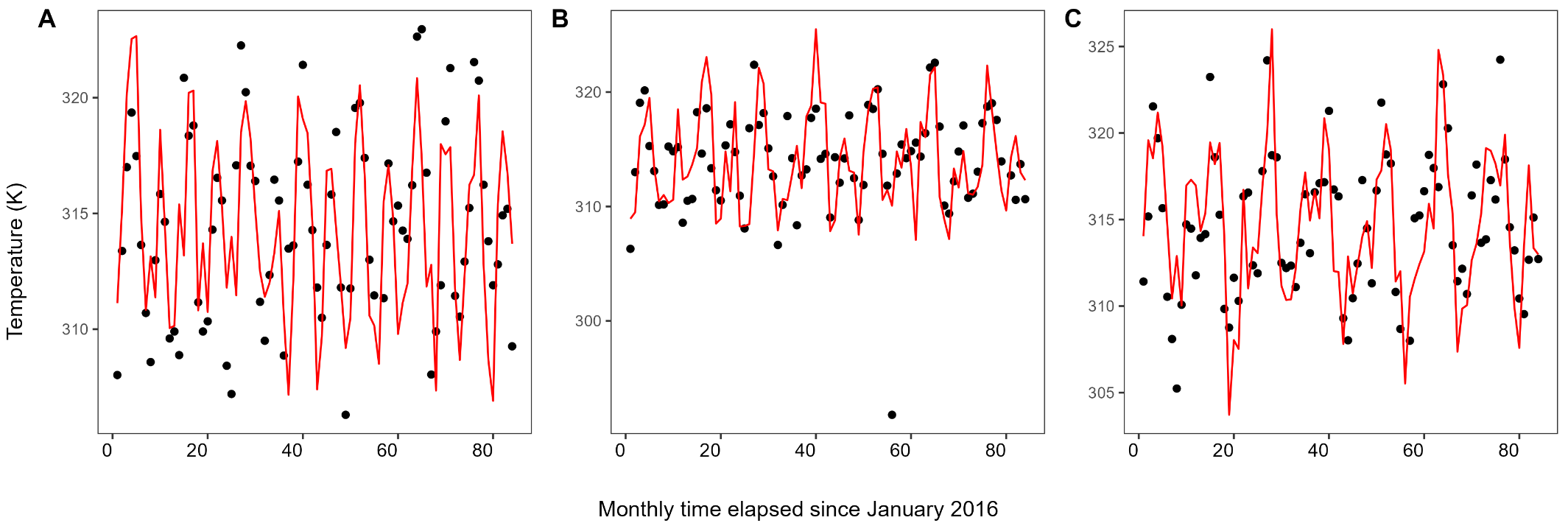


Figure S12. Observed temperature (in Kelvin) and fitted values at randomly selected pixels that contained missing values in Niger. The black dots are observed temperature values, and the red line shows the predicted values. The pixels were selected randomly among the ones that had at least one missing value.


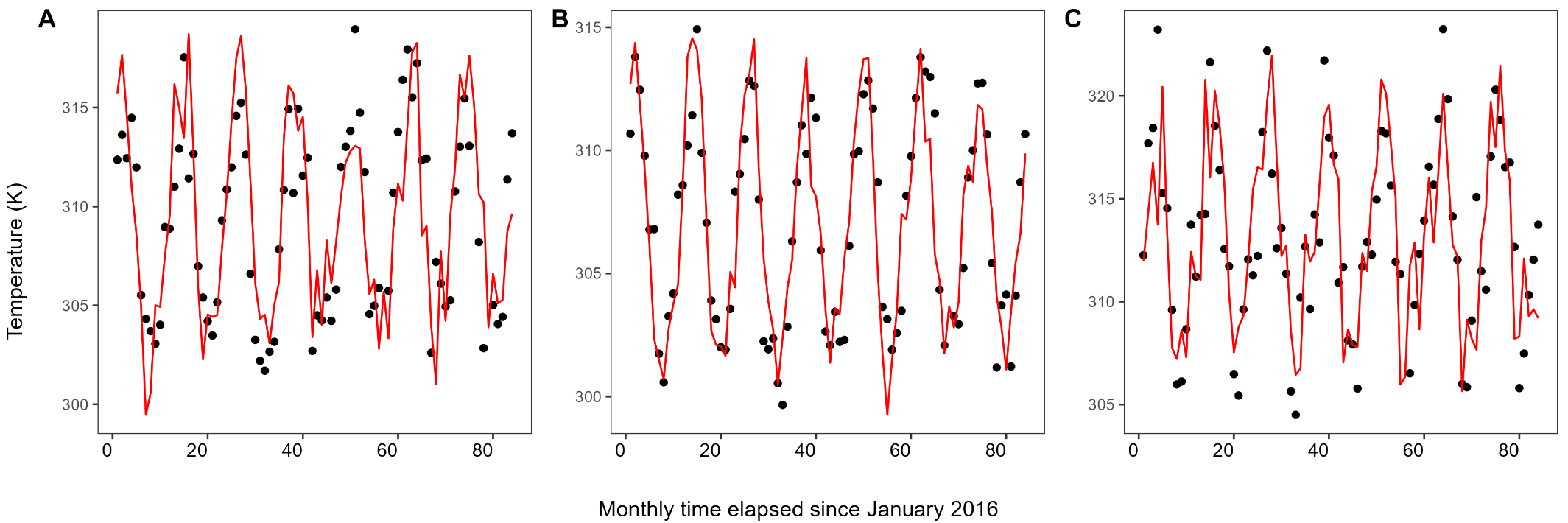


Figure S13. Observed temperature (in Kelvin) and fitted values at randomly selected pixels that contained missing values in Nigeria. The black dots are observed temperature values, and the red line shows the predicted values. The pixels were selected randomly among the ones that had at least one missing value.

**References**

1. Zhao B, Mao K, Cai Y, Shi J, Li Z, Qin Z, et al. A combined Terra and Aqua MODIS land surface temperature and meteorological station data product for China from 2003 to 2017. Earth Syst Sci Data. 2020;12.
